# Vitamin D3 Prevents the Deleterious Effects of Testicular Torsion on Testis by Targeting miRNA-145 and ADAM17: In Silico and In Vivo Study

**DOI:** 10.3390/ph14121222

**Published:** 2021-11-25

**Authors:** Doaa I. Mohamed, Doaa A. Abou-Bakr, Samar F. Ezzat, Hanaa F. Abd El-Kareem, Hebatallah H. Abo Nahas, Hosam A. Saad, Amir E. Mehana, Essa M. Saied

**Affiliations:** 1 Department of Clinical Pharmacology and Therapeutics, Faculty of Medicine, Ain Shams University, Cairo 11566, Egypt; 2 Department of Physiology, Faculty of Medicine, Ain Shams University, Cairo 11566, Egypt; doaaabou-bakr@med.asu.edu.eg; 3 Department of Histology and Cell Biology, Faculty of Medicine, Ain Shams University, Cairo 11566, Egypt; Samarezzat@med.asu.edu.eg; 4 Zoology Department, Faculty of Science, Ain Shams University, Cairo 11566, Egypt; Hanaafathy@sci.asu.edu.eg; 5 Zoology Department, Faculty of Science, Suez Canal University, Ismailia 41522, Egypt; hebatallah_hassan@science.suez.edu.eg (H.H.A.N.); amirelsayed@science.suez.edu.eg (A.E.M.); 6 Department of Chemistry, College of Science, Taif University, P.O. Box 11099, Taif 21944, Saudi Arabia; h.saad@tu.edu.sa; 7 Chemistry Department, Faculty of Science, Suez Canal University, Ismailia 41522, Egypt; 8 Institute for Chemistry, Humboldt Universität zu Berlin, Brook-Taylor-Str. 2, 12489 Berlin, Germany

**Keywords:** testis, testicular torsion detorsion, ischemia-reperfusion injury, histopathology, miRNA145, ADAM17, vitamin D3, apoptosis, sperm, molecular modelling

## Abstract

Testicular torsion (TT) is the most common urological emergency in children and young adults that can lead to infertility in many cases. The ischemia-reperfusion (IR) injury due to TT has been implicated in the pathogenesis of testicular damage. The main pathological mechanisms of contralateral injury after ipsilateral TT are not fully understood. In the presented study, we investigated the molecular and microscopic basis of ipsilateral and contralateral testicular injury following ipsilateral testicular torsion detorsion (T/D) and explored the possible protective role of vitamin D3. The biochemical analysis indicated that IR injury following T/D significantly decreased the activity of testicular glutathione peroxidase (GPx) enzyme, level of serum testosterone, serum inhibin B, and expression of testicular miRNA145, while increased the activity of testicular myeloperoxidase (MPO) enzyme, level of testicular malondialdehyde (MDA), level of serum antisperm-antibody (AsAb), and expression of ADAM-17. The histological and semen analysis revealed that torsion of the testis caused damages on different tissues in testis. Interestingly, administration of vitamin D3 prior to the IR injury reversed the deterioration effect of IR injury on the testicular tissues as indicated by biochemical and histological analysis which revealed normal appearance of the seminiferous tubules with an apparent decrease in collagen fiber deposition in both ipsilateral and contralateral testes. Our results revealed that the protective effect of vitamin D3 treatment could be attributed to target miRNA145 and ADAM17 protein. To further investigate these findings, we performed a detailed molecular modelling study in order to explore the binding affinity of vitamin D3 toward ADAM17 protein. Our results revealed that vitamin D3 has the ability to bind to the active site of ADAM17 protein via a set of hydrophobic and hydrophilic interactions with high docking score. In conclusion, this study highlights the protective pharmacological application of vitamin D3 to ameliorate the damages of testicular T/D on the testicular tissues via targeting miRNA145 and ADAM17 protein.

## 1. Introduction

Testicular torsion (TT) is the most common urologic emergency in children and adolescent males that can lead to infertility if left untreated [1]. Recent studies showed that 38% of males, with a history of torsion, has a sperm count of <20 million per mL [2]. The turning of the spermatic cord and spermatic tissues cause various biochemical and histopathological alterations which lead to testicular malfunction [3]. TT showed the ability to disrupt the blood flow to the testicular tissues which lead to ischemia [4]. When the blood circulation is resumed after an episode of sever ischemia, ischemia-reperfusion (IR) damage is ensued. After TT, the high levels of reactive oxygen species (ROS) formed lead to the loss of cell viability via lipid peroxidation in the cellular membrane, protein denaturation, and DNA damage [5,6]. Furthermore, TT causes recruitment of activated neutrophils to microvascular endothelium and the subsequent emigration and release of myeloperoxidase (MPO) enzyme, which catalyzes the generation of ROS [7]. The high levels of nitric oxide (NO) and tumor necrosis factors ((TNF)-α and interleukin (IL) 1-β) enhance the inflammation and the interaction of neutrophils with oxidative and nitrosative systems [8]. On the other hand, testicular detorsion (T/D), causes endure reperfusion in tissues, which lead to severe injury than ischemia [9]. Detorsion and reperfusion also lead to the production of various pro-inflammatory cytokines which cause testicular atrophy, apoptosis of germ cells, and destruction of spermatogenesis, and impair the function of Sertoli cells [3]. The ipsilateral testicular damage after spermatic cord torsion is well-reported, however, the status of the contralateral testis remains obscure. Although several studies indicated that the contralateral testis is not affected by unilateral torsion, others have advocated the opposite view [10,11].

The tumor necrosis factor alpha converting enzyme (TACE), also recognized as A Disintegrin And Metalloproteinase 17 (ADAM17), is a membrane-bounded protein that regulates the shedding of ectodomain and liberation of a number of transmembrane proteins from juxatamembrane [12]. The shedding process causes the activation or inhibition of downstream signaling and cellular reactions and is linked to a number of acute and chronic inflammatory disorders [13]. ADAM17 is expressed in germ cells, and its activity is necessary for the induction of apoptosis in the germ cells [14]. IR causes hypoxia and oxidative stress that triggers ADAM17 activation [15]. Therefore, pharmacological targeting of ADAM17 has been considered as a potential approach to prevent apoptosis in germ cells with TT [16]. MicroRNAs (miRNAs) are short single-stranded coding RNAs of 18–25 nucleotides in length that regulate various biological activities such as embryonic development, cell cycling, apoptosis and stress responses [17]. Emerging evidence has indicated that miR-145 represses the migration and invasion of several malignant tumor cells by targeting ADAM17 [18]. Doberstein et al. reported that ADAM17 negatively regulates miR-145 expression [19]. Transfection with miR-145 imitate inhibited cell growth and triggered cell cycle arrest in the G0/G1 phase by upregulating the phase regulators, p53 and p21 [20]. In mammalian spermatogenesis, minor changes in miRNA expression substantially affect the function of germ cells, such as impaired spermatogenesis and compromised fertility [21].

1, 25-Dihydroxy cholecalciferol (vitamin D3) is traditionally known for its action for maintaining and regulating calcium and phosphorus homeostasis [22]. Apart from its general impact on mineral metabolism, vitamin D3 acts as an important endocrine hormone, which plays a significant role in modulating reproductive processes [23]. In the last decade, the relationship between vitamin D3 and reproductivity has been progressively recognized, due to the presence of vitamin D receptors (VDR) in the reproductive organs [24]. Recent investigations revealed that treatment of vitamin D3 to aged rats delays the testicular aging by upregulating the activity of the antioxidant enzymes [25]. Moreover, vitamin D3 ameliorates the ROS scavenging pathway, partially through upstream regulation of glutathione reductase genes expression [26]. Testicular cells treated with vitamin D3 showed a marked increase in testosterone production and enhancement in the expression of mRNA of enzymes that involved in testosterone production [27]. Vitamin D3 induces the expression of several bioactive molecules that involved in the antioxidant defense system including GSH, GPx and SOD [28]. Moreover, vitamin D3 has a stimulatory effect to regulate the levels of Luteinizing Hormone (LH), and Follicle Stimulating Hormone (FSH) [29].

Based on the abovementioned facts, in the present study we investigated the role of miRNA-145 and ADAM17 pathways in the contralateral testicular injury after unilateral T/D. Furthermore, we explored the possible protective effect of vitamin D3 in the early targeting these pathways with subsequent amelioration of germ cell apoptosis.

## 2. Material and Methods

### 2.1. Animals and Grouping

All animal experiments were approved by the Institutional Animal Ethics Committee for Ain Shams University, Faculty of Medicine. Twenty-four male Wistar rats (weighing 150 to 200 g) were purchased from National Research Institute (Cairo, Egypt), and were housed in an animal house with temperature (22 °C) and lighting control (12 h (light)–12 h (dark) cycle). An adaptation period of 1 week was allowed before initiation of the experimental protocol. The rats were randomly distributed equally into four groups (*n* = 6):

- Control Naïve group.
- Sham operated group (SHAM); Rats subjected to all surgical steps as the other two groups except for torsion/detorsion.
- Testicular Torsion/Detorsion group (T/D): T/D group: Rats were subjected to 720° torsion for 2 h then detorsion with subcutaneously injection of sesame oil (as a vehicle to vitamin D3) for 30 days.
- TT Testicular T/D; vitamin D3 treated group (T/D; D3); Rats subjected to 720° torsion for 2 h then detorsion with subcutaneous injection of vitamin D3 in a dose of 500 IU/Kg/day (20), starting half an hour before detorsion, then given daily, 5 days/week, for 30 days.

### 2.2. Chemicals and Reagents

Vitamin D3 was obtained as Devarol ampoule, 5 mg/2 mL (200,000 IU/2 mL), supplied by CHEMIPHARM pharmaceuticals industries, Egypt. Sesame oil was supplied by El Hawag for Natural Oils Company, Egypt. 0.5 mL of devarol ampoule (50,000 IU) was dissolved in 99.5 mL sesame oil to reach final concentration of 500 IU/1 mL, injected subcutaneously as 0.1 mL/100 g. rat B.W.

### 2.3. Testicular Torsion/Detorsion Animal Model

After being anaesthetized with ether inhalation, rats were fixed on the table on their back, then the skin of the scrotum was disinfected with betadine solution, and all procedures were performed under sterile conditions. A left vertical paramedian incision was made on the scrotum and the left testis was exposed, manually rotated 720° clockwise (two cycles of full rotation) to perform torsion and fixed by clipping. Then, the scrotum was covered by a piece of cotton soaked with normal saline. After 2 h the left testis was exposed, detorted and placed back in its anatomical position. The scrotal incision was closed with 2/0 silk suture [30]. After 30 days, rats were overnight fasted and anaesthetized with pentobarbital in a dose of 40 mg/Kg B.W., Retroorbital samples were collected in a plain tube and centrifuged at 3000 rpm for 15 min. Then, serum was separated and stored at −80 °C for later determination of total testosterone, inhibin B and serum AsAb. The scrotum was reopened to extract the left (ipsilateral) and contralateral testis that was stored at −80 °C for later biochemical analysis of testicular malondialdehyde (MDA), myeloperoxidase (MPO), glutathione peroxidase (GPx), miRNA-145 and ADAM17 gene expression.

### 2.4. Biochemical Measurements

- Assessment of testicular endocrinal function: Assessment of testicular endocrinal function was performed by measuring the serum level of total testosterone using Steroid EIA (enzyme immunoassay)-Testosterone, ALKPR-BIO, France. Inhibin B was measured by rat specific inhibin B ELISA (enzyme linked immunosorbent assay) kit, My Bio Source, San Diego, CA, USA.
- Determination of testicular oxidative stress markers: Assessment of testicular oxidative stress markers was calorimetrically performed using MDA OxiSelect “TBARS; thiobarbituric acid reactive substances” assay kit, CELL BIOLABS, USA, and GPx assay kit, Cayman Chemical, Ann-Arbor, MI, USA.
- Assessment of testicular inflammatory response: Assessment of testicular inflammatory response was evaluated by measuring MPO using rat specific CLIA (chemiluminescent immunoassay) kit, Life Span Bio Sciences, Seattle, WA, USA.
- Estimation of immunological reaction: Assessment of immunological reaction was estimated by evaluation of serum AsAb using rat specific ELISA kit, Cube Biosystems, College Park, MD, USA.

### 2.5. Assessment of Apoptotic Process

- Testicular ADAM17 Expression

ADAM17 were determined by Sun Red (England) ELISA kits. Precoated wells with the captured antibodies were washed four times with the washing buffer. Standard or samples (100 μL) were added to each well in duplicate, covered, and incubated at RT for 2 h. The plates were then washed four times and 100 μL of the diluted antibody was added per well, covered, and incubated at RT for 2 h. 100 μL of streptavidin-HRP was added to each well and incubated for 30 min at RT for a proper colour development. The plates were washed and 100 μL of the substrate solution (3,3,5,5-tetramethylbenzidine, TMB) was added to each well and incubated for 30 min at RT. The reaction was terminated by adding 100 μL of the stop solution (H_2_SO_4_, 5%) to each well, and the developed color was recorded at 450 nm on a plate reader (Sanofi Diagnostics Pasteur, Lyon, France) [31].

ii. Assessment of miRNA-145 by Real time PCR

Tissue Homogenization: The tissue samples were suspended in a 2.0 mL screw captube, containing 200 uL of phosphate buffer saline (PBS), a single 5 mm stainless steel bead (Qiagen) was added to each tube and the samples were homogenized at maximum speed (30 Hertz) for 2 min using the Qiagen Tissue Lyser system. Following homogenization, the samples were spun for 1 min at maximum speed to reduce foaming, and the homogenate was then applied to the filter column for RNA extraction.

miRNA, and total mRNA extraction and purification: miRNA and total mRNA was extracted, using a miRNeasy Mini Kit (Qiagen, Hilden, Germany) according to the manufacturer’s protocol. Reverse transcription: cDNA was synthesized by reverse transcription reaction using miScript II RT Kit (Qiagen, Hilden, Germany).

miRNA expression analysis: The quantification of miR-145 levels was performed using the SYBR-Green fluorescent-based primer assay (hsa-miR-145: cat no: MS00003528); the Hs_RUN6-2_11; cat no: MS00033740 primer assay was used as housekeeper gene for normalization. The qPCR was performed in the 5-plex Rotor Gene PCR System (Qiagen, Hilden, Germany). The 20 uL reaction mixture/reaction consist of 2× QuantiTect syber green PCR mastermix, 10× miscript universal primer, 2 uL primer assay and 50 pg–3 ng cDNA. Both targets were amplified in duplicates for each sample. The thermal protocol consists of 15 min for HotStarTaq DNA Polymerase activation at 95 °C followed by 40 cycles of denaturation at 94 °C for 15 min, primer annealing for 30 s at 55 °C and extension at 70 °C for 30 s). The 2∆∆Ct method was conducted for the analysis of miR-145 expression levels, using RUN6 as an endogenous reference control for normalization purposes.

### 2.6. Histological Studies

(a) Light microscopic study

Testis from each rat were immediately fixed in aqueous Bouin’s solution and processed to obtain paraffin sections of 5 μm thickness. Paraffin sections were stained with H&E and Mallory’s trichrome stain [29].

(b) Transmission electron microscopic (TEM) study

Each testis was cut into small pieces about 1 mm^3^ and were rapidly fixed in 2.5% glutaraldehyde. Specimens were processed and embedded in Epon resin. Semithin sections (50 nm) were stained with toluidine blue. Ultrathin sections were cut and mounted on copper grids [32]. Specimens were examined and photographed using a JEM 1200 EXII, JEOL, Tokyo, Japan transmission electron microscope at the Regional Center for Mycology and Biotechnology, Al Azar University.

(c) Semen analysis study

The right epididymis was quickly removed from all rats. Each epididymis was macerated by scissors into four parts, then they were left in in a hollow plate containing 1 mL physiological saline. The tissue was left for 30–60 s to allow sperms leak from the tubules (the solution will tint whitish/grayish). The resulted fluid was handled as the semen. Sperm suspension was collected into an Eppendorf tube to perform the semen analysis and sperm cell count using Nigrosine-Eosin staining technique. Briefly, two drops of 1% aqueous Eosin Y were added to one drop of semen and were mixed with a wooden stirrer for 15 s. Then two drops of 10% aqueous Nigrosine were added and mixed well with a wooden stirrer. Thin smears were immediately made and were let air-dry. Nigrosine provides a dark background that make it easy to visualization of sperm. Live spermatozoa have white or faint pink heads, and dead spermatozoa have heads that are stained red or dark pink [30].

### 2.7. Morphometric Study

It was performed using the image analyzer Leica Q win V. 3 program installed on a computer in the Histology and Cell Biology Department, Faculty of Medicine, Ain Shams University. The computer was connected to a Leica DM2500 microscope (Wetzlar, Germany). Specimens from all groups were subjected to morphometric study. Measurements were taken from five different slides obtained from each specimen. Five different non-overlapping fields were examined from each slide to measure the following:

1. Mean diameter of seminiferous tubules (X20)
2. Mean thickness of germinal epithelium (X20)
3. Mean area percentage of collagen fibers in Mallory’s trichrome stained sections (X20)

For mean diameter of seminiferous tubules, rounded transversely cut tubules were selected and two diameters of each tubule, one perpendicular to the other, were measured and the average taken [33].

### 2.8. In Silico Molecular Modelling Study

The binding affinity of vitamin D3 to ADAM17 protein was investigated by in silico molecular docking. The molecular docking studies were performed using Molecular Operating Environment (MOE, 2015.10) software. In the Protein data bank (PDB), there are more than 23 crystal structure for ADAM17 co-crystallized with different substrates and inhibitors [34,35,36,37,38,39,40,41,42,43,44,45,46,47,48,49,50,51]. To deeply investigate the binding affinity of vitamin D3 toward ADAM17 protein, we have performed extensive molecular modelling studies for vitamin D3 toward the different available crystal structures of ADAM17 protein. The 3D-crystallographic structures of ADM17 protein co-crystallized with inhibitors (PDB code: *2ddf, 3l0v, 3ewl, 3kmc, 3kme, 3le9, 3o64, 2i47, 3e8r, 3edz, 3lgp, 3l0t, 1bkc, 2oi0, 3b92, 3lea, 2fv9, 2fv5, 3g42, 1zxc, 2a8h*) were downloaded from Protein Data Bank website (http://www.rcsb.org/pdb, accessed on 1 September 2021). The water molecules and unnecessary chains were deleted. The Protonate 3D protocol in MOE with default options was used to prepare the protonated 3D proteins for the docking studies. All minimizations were performed using MOE until the root mean square deviation (RMSD) incline of 0.05 kcal∙mol^−1^Å^−1^. The 2D structure of vitamin D3 was obtained from Chemdraw program. The Conf Search module in MOE was used to perform energy minimization and geometry optimization. MMFF94x force field was applied, and all the partial charges were automatically expressed [52,53,54]. Validation of the docking protocol was performed by re-docking the original co-crystallized substrate/inhibitor into the binding site of the protein structure in order to confirm the validity of the PDBs selected for the docking process. The Triangle Matcher placement protocol and London dG scoring function were applied in the validation protocol. After examining the protocol for the validation step, the protocol was then applied to dock vitamin D3 in the ADAM17 binding site to evaluate its binding affinity. The acquired results were evaluated, and the poses with high ligand-enzyme binding affinity were selected for energy estimation.

### 2.9. Statistical Study

Data were collected, revised then subjected to statistical analysis using one-way ANOVA performed by SPSS.21 program (IBM Inc. Chicago, IL, USA). Data were presented as mean ± standard deviation (SD) The significance of the data was determined by *p* value *p* > 0.05 is considered non-significant and *p* < 0.05 significant.

## 3. Results

### 3.1. Biochemical Analysis

#### 3.1.1. Effect of Testicular T/D and Vitamin D3 Treatment on Oxidative Stress Markers in Ipsilateral and Contralateral Testis

Frist, we have explored the effect of testicular T/D on oxidative stress markers by evaluating testicular GPx, MDA and MPO expression. As shown in Table 1, there was no significant difference between control naïve and sham group. On the other hand, testicular T/D caused a significant decrease (*p* < 0.05) in tissue GPx, while significantly increased (*p* < 0.05) the level of MDA and MPO. These results indicate that ischemic reperfusion injury following T/D decreased the protective GPx expression which leads to increase the production of lipid peroxides and testicular tissue damage. Furthermore, in response to ischemic reperfusion injury, while the recruitment of neutrophils to testicular tissue induces the release of MPO which triggers lipid peroxidation of the cell membrane, the release of MDA leads to protein denaturation, DNA damage and cell death. Next, we have investigated the effect of vitamin D3 on testicular GPx, MDA and MPO expression after T/D. The results revealed that vitamin D3 caused a significant (*p* < 0.05) reversal of the levels of oxidative stress markers to that of the control group. These results indicate that vitamin D3 has the ability to restore the antioxidant balance toward normal conditions and prevent testicular tissue damage by amelioration of lipid peroxidation of cell membrane.

#### 3.1.2. Effect of Vitamin D3 Treatment on Serum Testosterone, FSH, Inhibin B, Anti-Sperm Antibody in Testicular T/D Rat Model

To examine the endocrinal function of the testis and the immunological in response to testicular T/D, we have assessed the level of serum testosterone, FSH, serum inhibin B and AsAb. As shown in Table 2, testicular T/D caused a significant decrease (*p* < 0.05) in serum testosterone and inhibin B as compared to control naïve and sham group. While testicular T/D produced a significant increase (*p* < 0.05) in serum FSH and AsAb as compared to control naïve and sham group. The decreased testosterone level in T/D group indicated Leydig cell failure for steroidogenesis due to decrease in blood flow to testicular cells which caused necrosis and apoptosis of testicular tissue. The elevated FSH level could be related to a low testosterone level. Decreased serum Inhibin B in T/D group has been ascribed to an early arrest of spermatogenesis and loss of Sertoli cell function. Elevated serum level of AsAb could be explained by an immunological reaction and/or a blood testicular barrier damage following testicular T/D.

Furthermore, we investigated the endocrinal and immunomodulatory effect of vitamin D3 after testicular T/D. The results revealed that the treatment with vitamin D3 induced a significant (*p* < 0.05) reversal of the hormonal changes toward contralateral testicular protection with amelioration of ipsilateral testicular injury as compared to T/D group. There was no significant difference between the control naïve and sham group. These results demonstrated that vitamin D3 is able to restore the Leydig and Sertoli cell function and reduce the AsAb level which indicate that either immune reaction alleviation or restoration of testicular blood barrier integrity.

#### 3.1.3. Effect of Testicular T/D and Vitamin D3 Treatment on Testicular miRNA-145 and ADAM17

To gain insights into the molecular changes in response to testicular T/D, we have investigated the gene expression of testicular miRNA-145 and ADAM17. As shown in Table 3, testicular T/D caused a significant decrease (*p* < 0.05) in testicular miRNA-145, while significantly increased (*p* < 0.05) ADAM17 in ipsilateral and contralateral testes as compared to control naïve and sham groups. These results indicated that ischemic reperfusion injury induced upregulation of ADAM17 with down regulation of its epigenetic regulator miR-145. Both of ADAM17 and miR-145 play a key role in diverse biological processes like spermatogenesis and germ cell apoptosis. Furthermore, we evaluated the antiapoptotic effect of vitamin D3 following testicular T/D. The results revealed that vitamin D3 produced a significant (*p* < 0.05) reversal of molecular changes in ipsilateral and contralateral testes. There was no significant difference between the control naïve and sham group. These results indicate that vitamin D3 prevented the testicular tissue damage via targeting the molecular controller of germ cell apoptosis miRNA-145/ADAM17 pathway and this signifying its anti-apoptotic properties on testicular tissue.

#### 3.1.4. Effect of Testicular T/D and Vitamin D3 Treatment on Relative Testicular Weight in Ipsilateral and Contralateral Testis

In order to investigate the effect of testicular T/D on testes weight, we have evaluated the final body weight (FBW), relative testicular weight (RTW), absolute testicular weight (ATW) in all groups. As shown in Table 4, testicular T/D produced a significant decrease (*p* < 0.05) in both ATW&RTW in ipsilateral and contralateral testis as compared to control naive and sham group. The reduced ATW and RTW, respectively in T/D testicles could be explained by the reduced blood supply during the torsion period, as well as the oxidative stress and leukocytic infiltration following its detortion (as indicated by high testicular MDA and MPO levels). In addition, the autoimmune reaction against the testicular tissue could be also a factor, as indicated by the elevated serum AsAb. Furthermore, we investigated the effect of vitamin D3 on RTW after testicular T/D. The results revealed that vitamin D3 treatment produced significant (*p* < 0.05) restoration of testicular as compared to testicular T/D group. There was no significant difference between the control naïve and sham group. These results could be explained by the enhanced stimulatory effect of vitamin D3 on Leydig cells, possibly by elevation of FSH, and LH levels and/or its antioxidant effect.

### 3.2. Histological Analysis

In order to affirm our results, we have evaluated the histopathological changes for the testes tissues following testicular T/D. First, we examined the stained sections of both testis of sham group. The stained sections, semen analysis and transmission electron microscope (TEM) sections showed no difference from the control group.

#### 3.2.1. Light Microscopic Analysis

**Figure 1:**
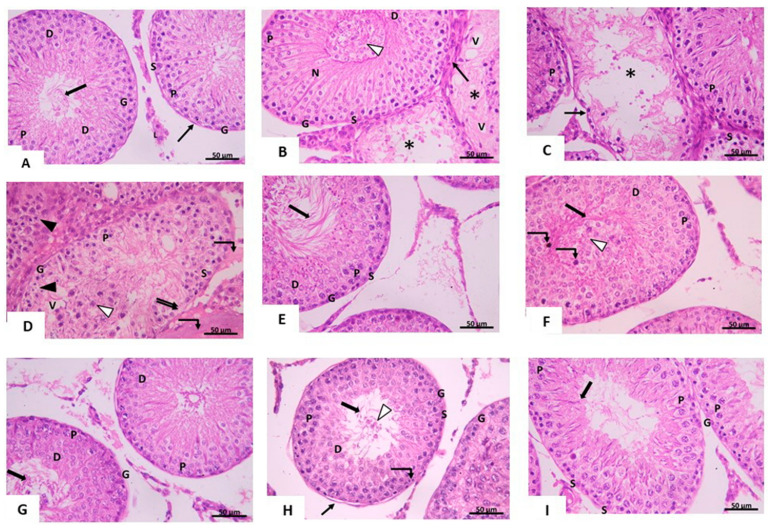
Photomicrograph of testis from (**A**) *control group* showing seminiferous tubules. Spermatogonia (G) and Sertoli cells (S) are resting on a regular basement membrane surrounded by myoid cell (↑). Primary spermatocytes (P), early spermatids (D) and spermatozoa within the lumen (thick arrow) can be seen. Clusters of interstitial Leydig cells (L) with their vesicular nuclei are observed between the tubules. H&E × 400. Scale bar: 50 µm. (**B**–**D**) *ipsilateral T/D group* Spermatogonia (G), primary spermatocytes (P), Sertoli cells (S) and early spermatids (D) are seen. (**B**) An apparently normal seminiferous tubule (N) is seen. Other tubules are seen distorted (*) with reduction of the thickness of the epithelial lining and lack of sperms in the lumen. Vacuolation (V) with apparent thickening of the basement membrane (↑) are noticed. Sloughing of spermatogenic cells is seen in the lumen of seminiferous tubules (∆). (**C**) Loss of spermatogenic epithelium lining the seminiferous tubule (*) and irregular distorted basement membrane is seen (↑). (**D**) area of loss of spermatogenic cells (↑↑), sloughed cells in the lumen (∆) and vacuolated (V) seminiferous tubules are seen. Homogenous acidophilic material (curved arrow) is seen in the interstitial space. Note the spermatogenic cells with vacuolated cytoplasm and small dark nuclei (▲). (**E**,**F**) *contralateral T/D group*: Spermatogonia (G), primary spermatocytes (P), Sertoli cells (S), early spermatids (D) and sperms (thick arrow) are seen. (**F**) obstruction of the lumen (∆) by sloughed spermatogenic cells. Distorted spermatogenic cells with deeply stained nuclei (curved arrow) are noticed. H&E × 400. Scale bar: 50µm. (**G**,**H**) *ipsilateral treated group* Spermatogonia (G), primary spermatocytes (P), Sertoli cells (S), early spermatids (D) and sperms (thick arrow) are seen. [1H] Few sloughed spermatogenic cells (∆) are seen in the lumen. Separation of spermatogenic cells from the basement membrane (↑) is seen. Notice the dividing cells (curved arrow) (**I**) *contralateral treated group:* Spermatogonia (G), primary spermatocytes (P), Sertoli cells (S) and sperms (thick arrow) are seen. H&E × 400. Scale bar: 50 µm.

Examination of H&E-stained sections of the control testis revealed closely packed seminiferous tubules separated by narrow interstitial spaces containing clusters of interstitial cells of Leydig with vesicular nuclei. Each seminiferous tubule was surrounded by a well-defined basement membrane and flattened myoid cells with flattened nuclei. Each tubule was lined with stratified germinal epithelium formed of spermatogonia, primary spermatocytes, early and late spermatids. Sertoli cells, resting on regular basement membrane, were seen between the spermatogenic cells. They had large pale vesicular nuclei. Spermatozoa were also seen within the lumen of seminiferous tubules (Figure 1A). Examination of sections of ipsilateral testis of T/D group (group III), showed loss of normal architecture of seminiferous tubules. Distortion with reduction of the thickness of the epithelial lining was noticed. Sometimes sperms were absent inside the lumen of the seminiferous tubules. Furthermore, sloughing of spermatogenic cells in the lumen of some seminiferous tubules was detected. Vacuolations were seen near the thickened irregular basement membrane with areas of loss of spermatogenic epithelium (Figure 1B,C). Homogenous acidophilic material was seen in the interstitial space. Some spermatogenic cells showed vacuolated cytoplasm and small darkly stained nuclei (Figure 1D). While examination of sections of the contralateral testis of the same group revealed obstruction of the lumen by spermatogenic cells and sperms. Distorted spermatogenic cells that appeared with deeply stained nuclei were detected in some seminiferous tubules (Figure 1E,F). Examination of sections of ipsilateral testis of vitamin D3-treated group (group IV) revealed separation and sloughing of few spermatogenic cells into the lumen of some tubules, while the rest of the tubules appeared nearly similar to control. Some dividing cells were also detected (Figure 1G,H). Sections of contralateral testis showed that most of the seminiferous tubules were nearly similar to the control group. Spermatogonia, primary spermatocytes, Sertoli cells and sperms were seen (Figure 1I). Our H&E-stained sections results revealed the damages caused by torsion of the testis and their effects on the different tissues. Furthermore, treatment of the testicular torsion by vitamin D3 improved these changes toward normal conditions.

**Figure 2:**
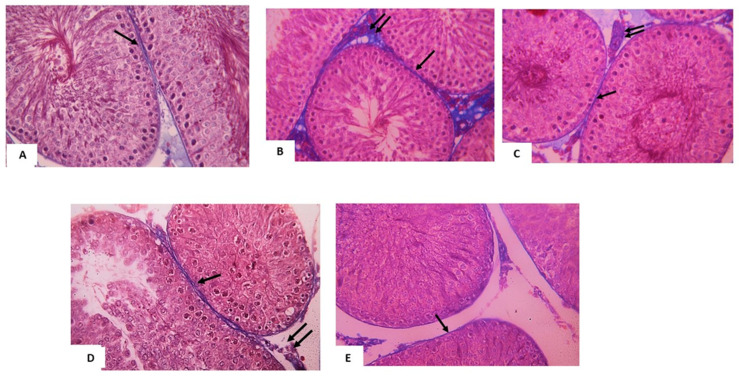
Photomicrographs of testis from (**A**) control group: few collagen fibers (↑) are seen surrounding seminiferous tubules. (**B**) ipsilateral T/D group: an apparent increase amount of collagen fibers around (↑) and in-between (↑↑) seminiferous tubule. (**C**) contralateral T/D group: Collagen fibers are seen around (↑) and in-between (↑↑) seminiferous tubule. (**D**) ipsilateral treated group: an apparent decrease amount of collagen fibers around (↑) and in-between (↑↑) seminiferous tubule as compared to the T/D group. (**E**) contralateral treated group: few collagen fibers (↑) are seen surrounding seminiferous tubules. Mallory‘s trichrome stain × 400. Scale bar: 50 µm.

Moreover, we investigated the collagen fibers in the testes using Mallory’s trichrome stain. The results showed that in the sections of the control group testis, few collagen fibers were seen around seminiferous tubules (Figure 2A). While in T/D group, a significant increase in the amount of collagen fibers was noticed around and in-between seminiferous tubules of ipsilateral and contralateral testes (Table 5 and Figure 2B,C). A significant decrease in collagen fibers deposition was noticed in both testes of vitamin D3 treated group (Table 5 and Figure 2D,E) as compared to group III. These results indicated that the testicular damage caused an obvious increase in collagen fiber deposition due to tissue fibrosis, and this increase restored to the normal level by the treatment with vitamin D3.

**Figure 3:**
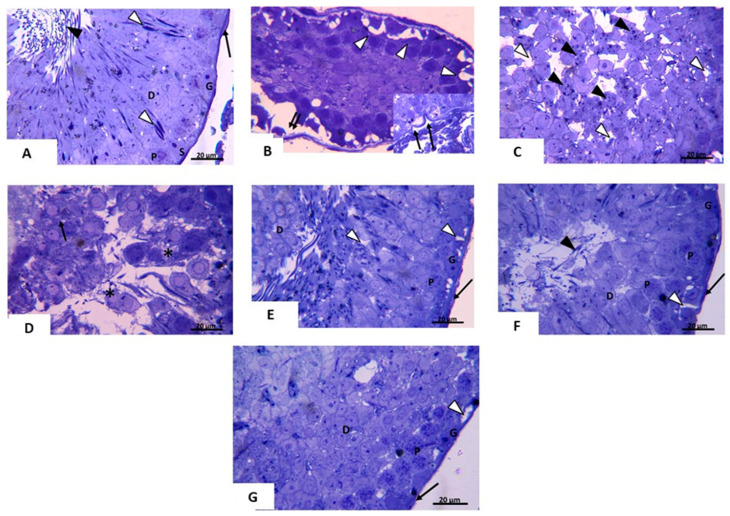
Photomicrograph of testis from (**A**) *control group:* the epithelium of seminiferous tubules is seen resting on a regular basement membrane and myoid cell (↑). Spermatogonia (G) are seen with ovoid nuclei, and Sertoli cell (S) with large vesicular pale nucleus. Primary spermatocytes (P) are the largest germ cells within seminiferous tubules. They have large nuclei containing strands of condensed chromosomes. Early spermatids (D) are small, rounded cells with small, rounded nuclei. Late spermatids (∆) are seen with diamond shaped irregular nuclei. Sperms (▲) are seen in the lumen Toluidine blue stain × 1000. Scale bar:20µm. (**B**–**D**) *ipsilateral T/D group*: Apparent reduction in the size of the seminiferous tubule. Spermatogenic cells are seen separated from the basement membrane (↑↑). Notice the multiple vacuolations (∆). Irregular basement membrane (↑) is seen (Inset). (**C**) Distorted spermatogenic cells are seen widely separated (∆). Cells with fragmented nuclei are also seen (▲). (**D**) sloughed cells are seen in the lumen of seminiferous tubule (*). ill-defined cell boundaries are noticed in most spermatogenic cells (↑) (**E**) *contralateral T/D group*: spermatogenic cells are seen resting on regular basement membrane surrounded by myoid cell (↑). Spermatogonia (G), primary spermatocytes (P) and spermatid (D) are seen. Vacuolations (∆) are seen between some germ cells. Toluidine blue stain × 1000. Scale bar: 20 µm. (**F**) *ipsilateral treated group*: spermatogenic cells rest on basement membrane (↑). Vacuolations (∆) are seen between spermatogonia (G). Primary spermatocytes (P), spermatids (D) and sperms (▲) are seen. (**G**) *contralateral treated group*: vacuolations near the basement membrane (∆) can be seen. Primary spermatocytes (P), early spermatids (D) and sperms (▲) are seen. Toluidine blue stain × 1000. Scale bar: 20 µm.

Next, semithin sections stained with toluidine blue were used to investigate the lesions that may occur in the cells and the nucleus of the testes. In toluidine blue stained sections of control group, the epithelium of seminiferous tubules was seen resting on regular basement membrane and flattened myoid cells. Spermatogonia were seen with rounded to oval nuclei, pyramidal shaped Sertoli cells with large vesicular nuclei were seen between adjacent spermatogenic cells. Primary spermatocytes were the largest germ cells found within seminiferous tubules; they were present in the middle layers of the germinal epithelium. They had a spherical outline and large nucleus with either thin threads or coarse clumps of chromatin. Early spermatids were small, rounded cells with small, rounded nuclei. Late spermatids with diamond shaped irregular nuclei were seen (Figure 3A). In Ipsilateral testis of T/D group, some seminiferous tubules were seen shrunken with vacuolations near the irregular thickened basement membrane. In some areas there were a separation of the germinal epithelium from the basement membrane (Figure 3B). Additionally, some tubules showed distortion of the regular arrangement of the spermatogenic cells which were separated by wide intercellular spaces. Cells with fragmented nuclei were also noticed (Figure 3C). Sloughed cells were seen in the lumen of seminiferous tubule and most spermatogenic cells appeared with ill-defined cell boundaries (Figure 3D). In the contralateral testis of T/D group spermatogenic cells were seen resting on regular basement membrane surrounded by myoid cell. Vacuolations were noticed between some germ cells (Figure 3E). In the sections of vitamin D3-treated group, ipsilateral testis showed spermatogenic cells resting on basement membrane. Primary spermatocytes, spermatids and sperms were seen. Few vacuolations were seen between spermatogonia. (Figure 3F). In contralateral testis, vacuolations were seen near the basement membrane. Primary spermatocytes, early spermatids and sperms were seen (Figure 3G). These results indicate that there is a damage occurred in the testis architecture due to the torsion, while the treatment with vitamin D3 improved these damages.

#### 3.2.2. Semen Analysis

**Figure 4:**
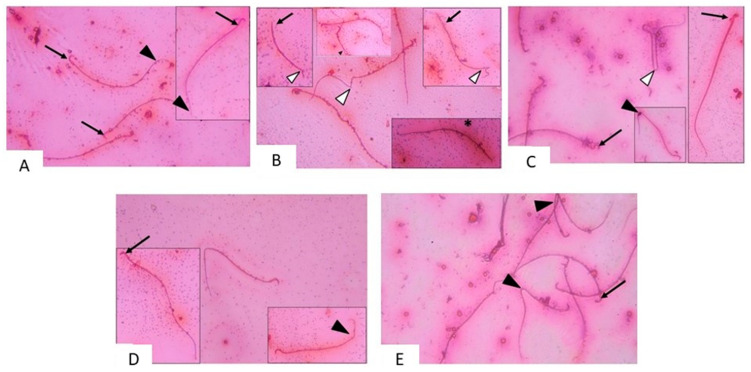
Photomicrographs of sperm smears from (**A**) control group: normal sperms are seen with hook shaped heads (↑) and tail (▲). (**B**) ipsilateral T/D group: abnormal sperms with detached head (↑), coiled tail (▲) kinked tails and sperms with abnormal tails (Δ) can be seen. Dead sperm is noticed (*) (**C**) contralateral T/D group: sperms with abnormal head (↑), coiled tail (▲), abnormal tail (Δ) can be seen. (**D**) ipsilateral treated group: sperms with normal hook shaped heads (↑) and tail bend toward the head (▲) are seen (**E**) contralateral treated group: sperms with normal hook shaped heads (↑) and coiled tail (▲) are seen. Nigrosine Eosin sperm smear × 400.

To gain insights into the reproductive function of the testis following testicular T/D, we have performed a detailed semen analysis. Toward this, Eosin-Nigrosin was used to stain sperm smears and assess the vitality of the sperm sample. While Nigrosin was used to increase the contrast between the background and sperm heads, leading to better visualization of sperms. In the control group, normal sperm with tail and hook shaped heads were seen in examination of Nigrosin-Eosin sperm smear (Figure 4A). In smears of ipsilateral testis of T/D group, abnormal sperms were frequently seen. Sperms with detached head, coiled and kinked tails were seen. Headless sperms which lack hook-shaped heads were noticed. Dead sperms could sometimes be detected (Figure 4B). While examination of smears of the contralateral testis of the same group showed sperms with abnormal head and abnormal tail (Figure 4C) These findings indicate that sperm structure and reproductive testicular function have been affected by the testicular torsion.

Furthermore, we evaluated the effect of vitamin D3 on semen production after testicular T/D. The results revealed that smears of ipsilateral testis of vitamin D3 treated group revealed apparent few abnormal sperms. Some sperms were seen with normal hook shaped heads and tail bend toward the head (Figure 4D). In contralateral testis of vitamin D3-treated group, few sperms with normal hook shaped heads and kinked tail were seen. Most of the sperms appeared more or less similar to the control (Figure 4E). These findings indicate that sperm structure and reproductive testicular function affected by the testicular torsion, while the treatment with vitamin D3 altered that damage and the testes nearly produced normal sperms.

#### 3.2.3. Transmission Electron Microscopic (TEM) Analysis

**Figure 5:**
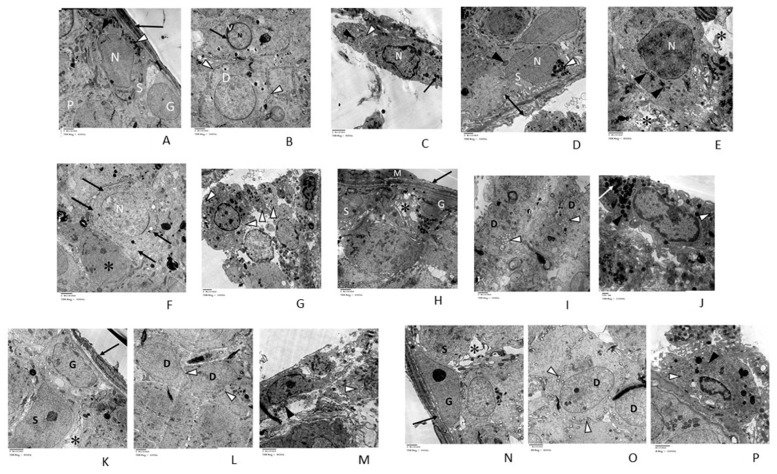
Electron micrographs of (**A**–**C**) *control group*: Columnar Sertoli cell (S) resting on a regular basement membrane which is surrounded by myoid cell (↑). Sertoli cell contains large pale euchromatic indented nucleus (N). It has complex lateral and apical infoldings. Its cytoplasm contains mitochondria (▲), and lipid droplets (Δ). Spermatogonia (G) and primary spermatocyte (P) with its large nucleus are seen. (**B**) Early spermatid (D) is seen containing large euchromatic nucleus and many peripherally arranged mitochondria with a clear matrix (Δ). Acrosomal vesicle (V) wrap around the nucleus (N). The anterior two-thirds of the nucleus is covered by the acrosome (↑). (**C**) Interstitial Leydig cell with large euchromatic nucleus (N) and a thin rim of peripheral dense chromatin. The cytoplasm contains sER (Δ), mitochondria (▲), and numerous electron-dense lipid droplets (↑). TEM (A,B × 6000) (C × 8000). (**D**–**G**) *ipsilateral T/D group*: (**D**) Sertoli cell (S) with its large-indented nucleus (N) is seen resting on an irregular apparently thick basement membrane (↨). The cytoplasm contains mitochondria (▲) and lipid droplets (Δ). (**E**) Primary spermatocyte is seen irregular with distorted nucleus (N) and elongated mitochondria (▲). Wide intercellular spaces (*) is seen. (**F**) Early spermatid with large nucleus (N) and loss of peripheral arrangement of mitochondria (↑) can be seen. Spermatogenic cell with ill-defined nuclear membrane (*) is also noticed. (**G**) Leydig cells are seen with dilated sER (Δ). TEM (D&G × 5000) (E × 8000) (F × 6000). (**H**–**J**) *contralateral torsion group*: (**H**) spermatogonia (G) and Sertoli cell (S) with its large-indented nucleus are seen resting on a basement membrane (↑) surrounded by myoid cell (M). Intercellular vacuolations (*) are seen. (**I**) early spermatids are seen with aggregated small vacuoles in their cytoplasm (Δ). (**J**) Leydig cell contains lysosomes and dilated sER (▲). TEM (H&I × 6000) (J × 15,000) (**K**–**M**) *ipsilateral treated group:* (**K**) Sertoli cell (S) with its large-indented nucleus and spermatogonia (G) are seen resting on regular basement membrane that is surrounded by myoid cell (↑). Vacuolations (*) are seen between cells (**L**) early spermatids (D) are seen with peripheral arrangement of mitochondria (Δ). (**M**) Leydig cells are seen with mitochondria (▲) and sER (Δ). TEM (K&M × 8000) (L × 6000). (**N**–**P**) *contralateral treated group:* (**N**) Sertoli cell (S) and spermatogonia (G) are seen resting on regular basement membrane that is surrounded by myoid cell (↑). Vacuolations (*) are seen between cells (**O**) early spermatids (D) are seen with peripheral arrangement of mitochondria (Δ). (**P**) Leydig cell is seen contain mitochondria (▲) and sER (Δ). TEM (N × 6000), (O × 8000), (P × 15,000).

To get a deep insight into our previous histopathological results, TEM analysis were performed to explore the effect of testicular T/D on cells and to characterize the degree of testicular damage. We have investigated the ultrastructure features of the cells by using ultrathin sections and transmission electron microscopic examination to the testis of control rats. The results indicated columnar Sertoli cell resting on a regular basement membrane surrounded by myoid cell. Sertoli cells showed large pale euchromatic indented nucleus with complex lateral and apical infoldings. Primary spermatocyte with its large nucleus and spermatogonia were seen (Figure 5A). Early spermatid was seen containing large euchromatic nucleus and many peripherally arranged mitochondria with a clear matrix. Acrosomal vesicle (V) wrapped around the nucleus. The anterior two-thirds of the nucleus was covered by the acrosome ((Figure 5B). Interstitial Leydig cell was seen with large euchromatic nucleus and a thin rim of peripheral dense chromatin. Smooth endoplasmic reticulum (sER), mitochondria, and numerous electron-dense lipid droplets were seen in the cytoplasm ((Figure 5C). Electron microscopic examination of ipsilateral testis of T/D group showed Sertoli cell with its large-indented nucleus resting on an irregular apparently thick basement membrane. The cytoplasm contained mitochondria and lipid droplets (Figure 5D). Primary spermatocyte was seen with irregular distorted nucleus and elongated mitochondria. Wide intercellular spaces were also noticed (Figure 5E). Loss of peripheral arrangement of mitochondria in early spermatid has been also observed. Some spermatogenic cells appeared with ill-defined nuclear membrane (Figure 5F). Leydig cells were seen with dilated sER, mitochondria and lipid droplets (Figure 5G). Sections of contralateral testis of T/D group revealed spermatogonia and Sertoli cell resting on a basement membrane surrounded by myoid cell. Intercellular vacuolations were seen (Figure 5H). Early spermatids were seen with multiple small vacuoles in their cytoplasm (Figure 5I). Leydig cell contained lysosomes and dilated sER (Figure 5J). In ipsilateral testis of treated group electron microscopic examination showed spermatogonia and Sertoli cell with its large-indented nucleus resting on regular basement membrane which was surrounded by myoid cell. Few vacuolations were seen between cells (Figure 5K). Early spermatids were seen with peripheral arrangement of mitochondria (Figure 5L). Leydig cells were seen with mitochondria and sER (Figure 5M).

While contralateral testis of vitamin D3-treated group showed spermatogonia resting on regular basement membrane that was surrounded by myoid cell. Few vacuolations were seen between cells (Figure 5N). Early spermatids were seen with peripheral arrangement of mitochondria (Figure 5O). Mitochondria and sER were seen in the cytoplasm of Leydig cells (Figure 5P). The ultrastructure changes in these sections indicated the damages occurred due to the testicular torsion and revealed that treatment with vitamin D3 could improve these changes to the normal state.

### 3.3. Histomorphometry Analysis

We supported our results by the histomorphometry analysis that allowed us to evaluate the diameters or thickness for different structures of the testis. As shown in Table 5, in T/D group (group III), both ipsilateral and contralateral testes showed a significant decrease in the mean diameter of seminiferous tubules and the mean thickness of germinal epithelium compared to the control group. Furthermoe, a significant increase was noticed in the mean area percentage of collagen fibers of both testes compared to the control group. In vitamin D3-treated group (group IV), both ipsilateral and contralateral testes revealed a significant increase in the mean diameter of seminiferous tubules, mean thickness of germinal epithelium and a significant decrease in the mean area percentage of collagen fiber compared to T/D group (group III). However, in the ipsilateral testis of group IV, there was a significant decrease in the mean diameter of seminiferous tubules and the mean thickness of germinal epithelium and significant increase in the mean area percentage of collagen fibers compared to the control group. While the contralateral testis of the same group showed a considerable decrease in the mean diameter of seminiferous, mean thickness of germinal epithelium and a non-significant increase in the mean area percentage of collagen fibers compared to the control group. These results confirmed our previous detailed findings that treatment of vitamin D3 restores the impairment that occurred due to the torsion in either ipsilateral or contralateral testicular parts.

### 3.4. In Silico Molecular Modelling Study

**Figure 6:**
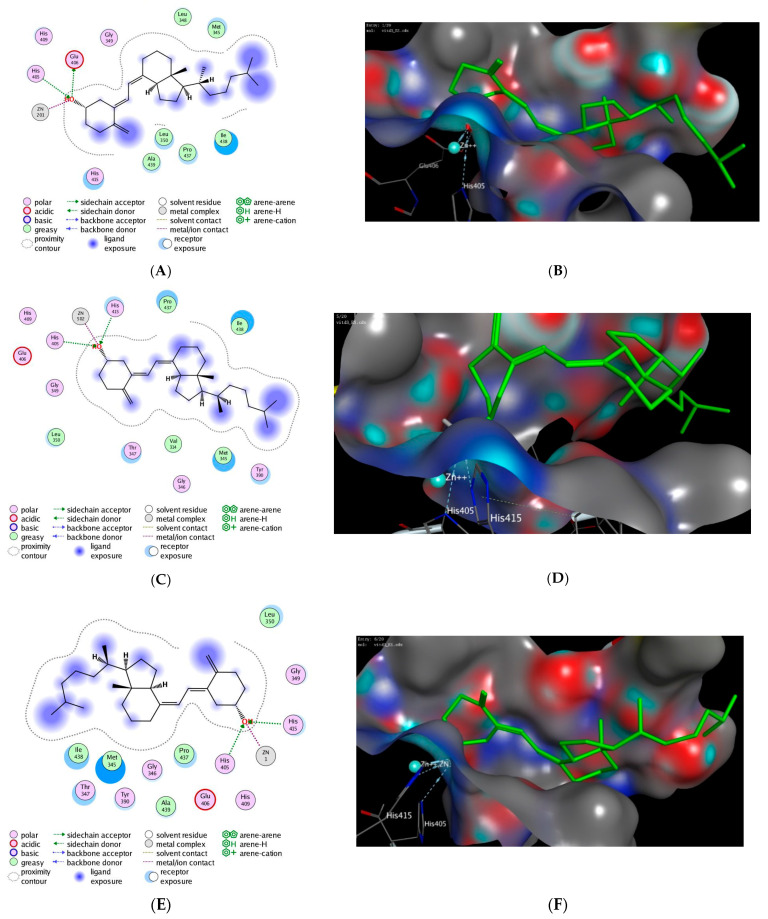
The 2D and 3D molecular docking interactions of the docked vitamin D3 (green in 3D interactions) with ADAM17 PDB: *1zxc* (**A**,**B**), *2b92* (**C**,**D**), and *3l0t* (**E**,**F**). The hydrogen bonds are illustrated as dotted blue arrows; (C atoms are colored green, and O red).

Molecular modelling approach has been considered as useful computational technique to assess the binding affinity of drug toward a target protein [55]. In order to gain insights into the mechanistic mode of action of vitamin D3 on ADAM17 protein, we have performed extensive molecular modelling studies to investigate the binding affinity of vitamin D3 toward the binding site of ADAM17 protein. In the Protein data bank (PDB), there are more than 23 crystal structures available for ADAM17 protein co-crystallized with different ligands [26,27,28,29,30,31,32,33,34,35,36,37,38,39,40,41,42,43]. Thus, molecular modelling approaches were performed for all available 3D crystal structure for ADAM17 protein, and results were extracted to be compared (Table 6). The best poses were selection based on the ability of vitamin D3 to possess the main interactions for binding to the protein. We first validated our docking protocol by re-docking the co-crystallized ligand to the binding site of ADAM17 protein. This step was successfully performed for all available PDB crystal structure for ADAM17 protein with a ~1.0–1.5 Å from the original co-crystalized ligand pose. In all cases, the selected docked poses showed the main interactions exist in the original ligand-protein interactions. The next step was to apply the validated protocol to investigate the ability of vitamin D3 to bind to the binding site of ADAM17 protein to evaluate its binding score and affinity. The scoring results of the docking process are shown in the Table 6. Our study revealed that vitamin D3 has a high binding affinity to bind to the active site of ADAM17 protein with high docking score via a set of hydrophobic and hydrophilic interactions (Figure 6, Table S1).

## 4. Discussion

Testicular torsion is an acute urological emergency affecting 1 in 4000 males aged <25 years which occurs due to a rotation of the spermatic cord [56]. Testicular torsion has been considered as a medical emergency that needs a quick interference and improper treatment as it may lead to male infertility. It is still controversial whether unilateral testicular T/D affects contralateral testis or not, although it is obvious that the protection of non-torsioned testis plays a vital role in fertility. Oxidative stress, inflammation and immunological responses have been implicated in the pathogenesis of contralateral testicular affection after unilateral testicular T/D [57]. In the current study, we investigated the pathogenesis of contralateral testicular affection after unilateral testicular T/D and the possible role of vitamin D3 on ipsilateral and contralateral testicular injury.

Toward these aims, the rats were subjected to 2 h of left TT followed by detorsion. TT caused an ischemic purple appearance in the ipsilateral testis. After detorsion, the testis appeared normal in color, suggesting that the testis is still viable. However, it was observed that unilateral testicular T/D produced pronounced injury in the ipsilateral and contralateral testis one month after detorsion, which was accompanied by significant decreases in ATW and RTW in T/D testicles, respectively. This could be attributed to a reduction of the blood supply during the torsion period [57], and to the overexpression of ROS which can damage lipids, proteins, and DNA, resulting in cellular dysfunction [58]. Our histomorphometry analysis results demonstrated that the decrease in the weight could be related to the decrease in the mean diameter of seminiferous tubules, and the mean thickness of germinal epithelium in the testicular T/D group which caused a decrease in the ATW and RTW. These results are in agreement with the study from Osemlak et al. which showed that exposure to testicular torsion caused a significant loss to the testicular weight due to a decrease in Sertoli cell number [59]. Moreover, our histological results showed that the normal architecture of the seminiferous tubules was lost together with distortion and reduction of the thickness of the epithelial lining with sloughing of the spermatogenic cells. The contralateral testis also showed nearly the same changes. Ozturk et al. demonstrated that the effects in germ cells layer due to testis torsion could be attributed to a high lipid peroxidation of the polyunsaturated fatty acids in germ cell membranes [60]. These findings may also explain the observed decrease in the ATW and RTW.

Next, we have performed a full semen analysis which showed abnormal sperms from both contralateral and ipsilateral testis. Indeed, most of sperms appeared with detached head, coiled and kinked tails, or as headless sperms which lack hook-shaped heads. In addition, dead sperms were also detected. In agreement with our findings, Jacobsen et al. recently reported a decrease in semen quality due to TT, together with the contralateral testis being unable to compensate adequately with increased spermatogenesis [61].

We also reported that the testicular T/D produced a significant decrease in serum testosterone and inhibin hormones concentration, while a noticeable increase was observed in serum FSH concentration and AsAb levels. The observed contralateral testicular damage could be attributed to the hormonal and immunological response to unilateral TT, which was indicated by the significant decrease in serum inhibin B and increased AsAb serum concentration [62].

Several studies demonstrated that TT caused a disturbance in spermatogenesis process that can be associated with a decrease in testosterone secretion. In addition, a significant decrease in sperm count and motility were observed in the torted testis [53,63]. Romeo et al. showed that the testicular torsion causes a dysfunction in both exocrine and endocrine of the testis, which may explain the observed impairment of the hormones level in our study (decrease in the testosterone and inhibin hormones and increase in FSH levels) [64]. Smith and Walker showed also that the malfunction of steroidogenesis in Leydig cells reduced the spermatogenesis process by targeting serum testosterone hormone [65]. Further studies indicated that the decrease in the blood flow to the testicular cells caused impairment in the spermatogenesis process and a significant decrease in the testosterone concentration, which resulted in apoptosis to the germ cells [66,67].

Inhibin B hormone has been considered as a direct marker of Sertoli cell function and indirect marker of spermatogenesis. Early arrest of spermatogenesis or a diminution of germ cells have been reported to lower the concentration of this hormone [68]. Recently, it was noticed that TT resulted in the reduction of inhibin B hormone which was associated with destruction of Sertoli cells [59]. Furthermore, Fu et al. suggested that the increase in serum inhibin B could indicate the degree of testicular injury [69]. Our results are in agreement with the study by Semercioz et al. which showed that the serum inhibin B levels suppressed after unilateral testicular T/D, suggesting contralateral testicular damage [70]. The observed elevation of serum AsAb levels could be attributed to collapse of the blood-testis barrier that resulted due to torsion of the ipsilateral spermatic cord which derived an immunological progression where AsAbs formed. The high serum AsAb levels has been also reported to significantly reduce the sperm motility and sperm concentration, which was well observed in our semen analysis results [61,71]. Based on these facts, AsAb and inhibin B levels could be considered as valuable markers for examining testicular function and after testicular T/D.

Although reperfusion is vital to the persistence of ischemic tissue, it leads to other cellular damage [72]. After testicular detorsion, ROS formed in these tissue causing injuries in the testicular lipids, proteins, carbohydrates and DNA, which leads to germinal cell apoptosis [73]. GPx is one of the antioxidant enzymes that aids in the conversion of ROS into less reactive species. MDA is the product of lipid peroxidation and has been considered as a respectable marker of free radical mediated-impairment and oxidative stress. Moreover, MPO is an enzyme found in neutrophils that mediates the infiltration of neutrophil to the tissues via transformation of the hydroperoxides into free radicals, initiating lipid peroxidation [60]. After reperfusion, there is imbalance between ROS and the antioxidant defense system. The sperms are sensitive to the ROS and mainly to lipid peroxidation due to their high content of polyunsaturated fatty acids in their plasma membrane. Furthermore, it was reported that IR caused formation of free radicals that lead to oxidative stress in tissues causing impairment of spermatogenesis [3,60]. In consistence with previous studies, we observed that the markers of oxidative stress (MDA, MPO enzymes) were significantly increased in the activity, while the activity of GPx enzyme was significantly decreased in both ipsilateral and contralateral testicular tissue. These results may be attributed to the formation of ROS which affects the antioxidant defense system and causes a decrease in the activity of GPx enzyme, as well as trigger the lipid peroxidation causing increase in the MDA concentration. Moreover, the infiltration of the neutrophil to the testicular tissue can cause an increase in the activity of MPO enzyme. Arena et al. reported that the damage in contralateral testicular could be associated with the interruption of blood-testis barrier via formation of ROS [74]. In addition, Turner and Lysiak demonstrated that reperfusion of the ischemic tissue stimulates the formation of ROS which resulted in an altering in the permeability and interrupting cell integrity [75]. On the other hand, reperfusion may be valuable for the ipsilateral testis by avoiding ischemia-induced apoptosis and necrosis. Wei et al. revealed that unilateral testicular IR caused infiltration of neutrophils to tissue and released MPO and MDA which led to a significant suppression in spermatogenesis in the ipsilateral testes [5].

ADAM17 activity is vital to stimulate apoptosis in physiological conditions. IR related to TT could induce activation of ADAM17 and shedding of TNFα as main feedback to hypoxia and oxidative stress [67,76]. Apoptosis is vital for normal spermatogenesis process in the testes of adults and for the development of different testicular disorders including undescended testes and torsion of the testes [77]. The TT and the release of TNF- α from Sertoli cells have been associated with germ cells apoptosis [16,78]. Furthermore, Meštrović et al. reported that the cell death in the non-torsioned side occurred via inflammatory and hormonal pathways, as well as, through miR145 and ADAM 17 pathways [79]. In our study, the apoptotic marker ADAM17 was significantly elevated, while its epigenetic regulator miR145 was significantly decreased in the contralateral testicular tissue after unilateral TT, suggesting germ cells apoptosis. Our results are in consistence with previous studies which showed that upregulation ADAM17 protein may induce the apoptosis to germ cell by activation of caspases and fragmentation of DNA [14,16,80].

In the male reproductive system, miRNAs have critical roles in different processes of spermatogenesis by mediating the expression of their targets [81,82]. Liu et al. demonstrated that miR145 could significantly suppress the expression of ADAM17 protein in hepatocarcinoma [18]. Based on these facts, miR145 could induce tumor cell apoptosis in TT through mediating the expression of certain apoptotic genes including ADAM17 gene expression. To the best of our knowledge, this is the first report which exclusively explored the role of ADAM17 protein and its epigenetic regulator miR145 in mediating the process of germ cell apoptosis in the contralateral testis after unilateral testicular T/D.

Vitamin D, (particularly the active form, 1,25 dihydroxy vitamin D3), is essential for human metabolism [83]. Vitamin D treatment reduces IR complications after a myocardial infarction by exerting anti-inflammatory and anti-apoptotic effects [84]. Furthermore, Yao et al. recently demonstrated the expression of vitamin D3 receptors in cells of the genital tract, such as spermatogonia [85]. These results suggest a role of vitamin D3 in spermatogenesis and sperm maturation in rats. These findings encouraged us to investigate and detect the efficacy of vitamin D3 on IR injury after testicular T/D.

**Figure 7:**
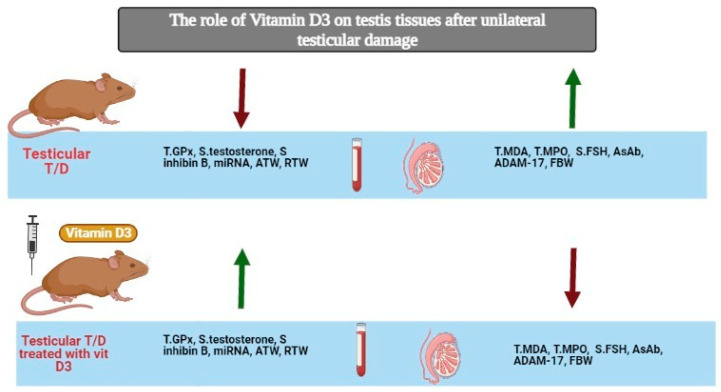
The role of vitamin D3 on testis tissues after unilateral testicular damage.

In our investigations, we reported that administration of vitamin D3 prior to the IR process has beneficial effects on oxidative damage of the testes. As summarized in Figure 7, administration of vitamin D3 revealed significant improvement in RTWwith significant increase in serum testosterone and inhibin B and decreased serum FSH which was associated with significant improvement in semen analysis profile, and induction of immunomodulatory effect via decreasing the production of AsAbs. Furthermore, vitamin D3 showed anti-apoptotic effects by decreasing ADAM17 gene expression and increasing its epigenetic regulator miR-145. These results were further confirmed by the histopathological studies and semen analysis profile which revealed normal appearance of the seminiferous tubules with apparent decrease in collagen fiber deposition in both ipsilateral and contralateral testes. The semen analysis after vitamin D3 treatment showed that the ipsilateral and contralateral treated testes contain few abnormal sperms and most of the sperms appeared more like the control group.

Vitamin D3 was shown to have a significant effect in improving sperm motility and morphology [86]. Alzoubi et al. reported that low serum vitamin D3 levels attenuated sperm parameters, while supplementation of vitamin D3 significantly increased the proportion and count of progressively motile sperm, as well as the testosterone concentration [87]. Furthermore, it was reported that vitamin D3 is directly involved in the development of the human male testicular androgenic system, increased testosterone production as well as the expression of mRNA for enzymes involved in androgen production and their precursors [88]. Since the testicular antioxidant capacity, apoptosis and proliferation are mainly regulated by testosterone hormone, Jeremy et al. hypothesized that vitamin D3 mediated-testosterone synthesis could be involved in the regulation of germ cell apoptosis and maintenance of antioxidants system in testicular tissue [24]. In agreements with our findings, Cito et al. showed that vitamin D3 was able to increase the serum inhibin B in oligozoospermic males, which reveals the clinical importance of vitamin D3 supplements in vitamin D-deficient infertile male subjects [89]. The observed decrease in AsAb levels after vitamin D3 treatment is in line with a recent study which showed that the immunomodulatory and immunosuppressive properties of vitamin D3 suppressed the AsAb production via restoration of testicular blood barrier integrity [90]. It has been also demonstrated that vitamin D3 treatment significantly decreased the lipid peroxidation and increased the activities of GPx in D-gal-induced aged testis. These results suggest that vitamin D3 mediates the protective antioxidant status in aged testis and might be responsible for the decrease in apoptosis and the increase in proliferation of germ cells [91]. Taken together, the presented findings suggest that vitamin D3 significantly decrease the stress biomarkers such as, MDA and MPO in ipsilateral and contralateral testes through antioxidative pathway. In addition, the antiapoptotic effect of vitamin D3 in testicular could be attributed to its ability to increase the expression of miR-145 and downregulate ADAM17 expression.

Furthermore, we have carried out extensive molecular modelling studies to get insights the mode of action of vitamin D3. As shown in Table 6, vitamin D3 showed a high binding affinity to bind to the active site of ADAM17 protein with high docking score by forming a set of hydrophobic and hydrophilic interactions. The key amino acid residues for interaction in the active site of ADAM17 protein are His415 and His405 residues, and these interactions are the key interactions commonly available between the different ligands and the protein in all available PDBs [92]. Under validated docking conditions, vitamin D3 showed the ability to make these interactions with high to moderate binding score (Figure 6, Table 6). Interestingly, vitamin D3 was also able in all poses to recognize and bind to Zn^+2^ ion in the binding site of the protein, which is also key interaction residue in all PDBs. In addition, vitamin D3 showed in some binding poses the ability to form additional hydrogen bonding with additional residues in the active site of ADAM17 protein. For instance, the energetically most favorable docked pose of vitamin D3 showed an extra hydrogen bonding with Glu406 residue (PDB, *2a8h*), with His409 residue (PDB, *3kme*, *3l0t*, *3g42*), or with Asp313 residue as shown with PDB, *3le9* (Figure 6, Table 6). The negative value of binding score reveals that the interaction between for vitamin D3 and ADAM17 protein was thermodynamically favorable in all cases. The docked poses analysis also demonstrated the role of the hydrophobic interactions in enhancing the binding affinity of vitamin D3. Indeed, vitamin D3 showed hydrophobic contacts between the hydrocarbon moiety of the scaffold and the hydrophobic residues at the protein cavity (Figure 6, Table 6). Overall, our molecular docking studies indicate that the effect of vitamin D3 on TT could be attributed to its ability to bind to the active site of ADAM17 protein.

## 5. Conclusions

In the present study, we showed that IR injury following T/D caused different changes in the biochemical and histological parameters, as well as in the apoptotic process and expression of some hormones. Our study revealed that IR injury caused upregulation of ADAM17 expression, while downregulated its epigenetic regulator miRNA145, suggesting a key role in diverse biological processes like spermatogenesis and germ cell apoptosis. The histological and histomorphological analysis indicated a damage in the testicular tissue as well as the sperm structure. Administration of vitamin D3 demonstrated beneficial and protective effects on the oxidative damage of the testes of patients with testicular torsion. Vitamin D3 treatment caused a significant increase in RTW, testosterone and inhibin B. The anti-apoptotic activity of vitamin D3 was confirmed by the histopathological studies and semen analysis profile, which revealed a normal appearance of the seminiferous tubules with an apparent decrease in collagen fiber deposition in both ipsilateral and contralateral testes. Furthermore, vitamin D3 significantly decreased stress markers such as MDA and MPO in ipsilateral and contralateral testes, probably through its anti-oxidative properties. Finally, our molecular docking study revealed the ability of vitamin D3 to bind to the active site of ADAM17 with high binding score. Collectively, our study demonstrated that the deterioration in the biochemical and histopathological parameters caused by IR injury could be reversed and ameliorated by the administration of vitamin D3. Accordingly, we hypothesize that the early administration of vitamin D3 to patients with unilateral testicular torsion could promptly protect the contralateral testicular affection and attenuate the unilateral testicular damage. Further studies should be conducted in future to investigate efficacy of vitamin D3 on the count, motility and viability of sperms after testicular torsion/detorsion. Moreover, the effect of vitamin D3 on the oxidative stress, hormonal disturbance, inflammatory and apoptotic pathways should be explored.

## Figures and Tables

**Table 1:** Effect of testicular T/D and vitamin D3 treatment on oxidative stress markers in ipsilateral and contralateral testis.

Parameter	Control Group	Sham Group	T/D Group	T/D (Vitamin D3) Group
T.GPx.I	1.812 ± 0.06534	1.793 ± 0.07967	0.1715 ± 0.08693 ^ab^	1.525 ± 0.1369 ^abc^
T.GPx.C	1.823 ± 0.2111	1.702 ± 0.1143	0.2933 ± 0.08824 ^ab^	1.142 ± 0.0332 ^abc^
T.MDA.I	255.7 ± 125.3	268.8 ± 127.9	2635 ± 238.2 ^ab^	1231 ± 221.3 ^abc^
T.MDA.C	240.5 ± 50.23	238.3 ± 44.35	436.2 ± 25.16 ^ab^	271.2 ± 39.28 ^bc^
T.MPO.I	30.32 ± 2.345	36.72 ± 3.706	95.90 ± 3.484 ^ab^	74.95 ± 5.430 ^abc^
T.MPO.C	29.55 ± 1.456	33.23 ± 1.564	55.75 ± 3.653 ^ab^	36.48 ± 2.373 ^abc^

Data are presented as mean ± SD. ^a^ *p* < 0.05 versus control group, ^b^ *p* < 0.05 vs. sham group, ^c^ *p* < 0.05 T/D group (*n* = 6). T.GPx.I: testicular glutathione peroxidase in ipsilateral testes, T.GPx.C: testicular glutathione peroxidase in contralateral testes, T.MDA.I: testicular malondialdehyde in ipsilateral testes, T.MDA.C: testicular malondialdehyde in contralateral testes, T.MPO.I: testicular myeloperoxidase in ipsilateral testes, T.MPO.C: testicular myeloperoxidase in contralateral testes.

**Table 2:** Effect of testicular T/D and vitamin D3 treatment on serum testosterone, FSH, inhibin B, and anti-sperm antibody.

Parameter	Control Group	Sham Group	T/D Group	T/D (Vitamin D3) Group
S. testosterone	138.5 ± 6.345	134.3 ± 5.982	64.40 ± 5.168 ^ab^	119.4 ± 3.838 ^abc^
S. FSH	0.7700 ± 0.1674	0.6500 ± 0.1378	2.212 ± 0.1429 ^ab^	1.717 ± 0.1472 ^abc^
S. inhibin B	40.34 ± 3.453	36.85 ± 2.359	22.93 ± 1.749 ^ab^	35.68 ± 2.982 ^abc^
Anti-sperm antibody	0.8012 ± 0.07623	0.7483 ± 0.06853	1.750 ± 0.1378 ^ab^	0.8083 ± 0.1209 ^abc^

Data are presented as mean ± SD. ^a^ *p* < 0.05 versus control group, ^b^ *p* < 0.05 vs. sham group, ^c^ *p* < 0.05 T/D group (*n* = 6). S. FSH: serum follicle stimulating hormone.

**Table 3:** Effect of testicular T/D and vitamin D3 treatment on testicular miRNA145 and ADAM 17 gene expression.

Parameter	Control Group	Sham Group	T/D Group	T/D (Vitamin D3) Group
T.micRNA145.I	40.23 ± 1.675	35.70 ± 1.953	13.30 ± 4.763 ^ab^	20.98 ± 9.640 ^abc^
T.micRNA145.C	43.56 ± 2.342	32.40 ± 2.605	19.73 ± 2.115 ^ab^	27.97 ± 3.881 ^abc^
T.ADAM17.I	1.950 ± 0.1912	2.250 ± 0.1871	8.100 ± 0.6356 ^ab^	3.252 ± 0.4450 ^abc^
T.ADAM17.C	2.119 ± 0.1366	2.133 ± 0.1366	3.517 ± 0.3061 ^ab^	2.465 ± 0.3252 ^abc^

Data are presented as mean ± SD. ^a^ *p* < 0.05 versus control group, ^b^ *p* < 0.05 vs. sham group, ^c^ *p* < 0.05 T/D group (*n* = 6). T.micRNA145.I: testicular micRNA 145 in ipsilateral testes, T.micRNA145.C: testicular micRNA 145 in contralateral testes, T.ADAM17.I: testicular ADAM 17 in ipsilateral testes, T.ADAM17.C: testicular ADAM 17 in contralateral testes.

**Table 4:** Effect of testicular T/D and vitamin D3 treatment on relative testicular weight of ipsilateral and contralateral testis.

Parameter	Control Group	Sham Group	T/D Group	T/D (Vitamin D3) Group
FBW	150.09 ± 5.23	149.10 ± 4.23	158.65 ± 2.83	165.23 ± 6.45 ^a,b^
ATW-I	0.93 ± 0.12	0.86 ± 0.05	0.58 ± 0.03 ^a,b^	0.72 ± 0.08 ^a,b,c^
RTW-I	0.66 ± 0.091	0.53 ± 0.082	0.29 ± 0.045 ^a,b^	0.40 ± 0.094 ^a,b,c^
ATW-C	0.100 ± 0.03	0.91 ± 0.01	0.63 ± 0.04 ^a,b^	0.86 ± 0.02 ^a,b,c^
RTW-C	0.75 ± 0.056	0.68 ± 0.064	0.39 ± 0.043 ^a,b^	0.64 ± 0.075 ^a,b,c^

Data are presented as mean ± SD. ^a^ *p* < 0.05 versus control group, ^b^ *p* < 0.05 vs. sham group, ^c^ *p* < 0.05 T/D group (*n* = 6). FBW: final body weight, ATW-I: absolute testicular weight of ipsilateral testes, RTW-I: relative testicular weight of ipsilateral testes, ATW-C absolute testicular weight of contralateral testes, RTW-I: relative testicular weight of contralateral testes.

**Table 5:** Diameter of seminiferous tubule, thickness of germinal epithelium, area percentage of collagen fibers in different rat groups (mean ± SD).

	Mean Diameter of Seminiferous Tubules (µm)	Mean Thickness of Germinal Epithelium (µm)	Mean Area % of Collagen Fibers
Control group	250.83 ± 2.04	74.50 ± 2.58	6.50 ± 1.76
Testicular T/D group (Ipsilateral testis)	212.00 ± 2.75 *	47.16 ± 2.93 *	14.50 ± 1.87 *
Testicular T/D group (Contralateral testis)	221.83 ± 4.70 *	59.00 ± 2.36 *	13.50 ± 2.42 *
Vit. D 3 treated group (Ipsilateral testis)	238.16 ± 2.92 *▲	66.16 ± 2.92 *▲	9.66 ± 1.63 *▲
Vit. D 3 treated group (Contralateral testis)	246.80 ± 3.96 ▲	72.20 ± 2.28 ▲	8.28 ± 1.11 ▲

* Significant change compared to control, ▲ significant change compared to torsion group.

**Table 6:** Scores and interactions of the docking process of vitamin D3 in ADAM17 binding sites.

PDB	Docking Score (kcal/mol)	Interactive Residues	
		Hydrophilic Interactions	Hydrophobic Interactions
*2ddf*	−13.63	Zn^+2^, His415	Pro437, Ile438, Ala439, Met345, Leu359
*3l0v*	−10.71	Gly346, His415	Ile438, Pro437, Ala439, Met345, Leu350, Ala351, Pro356, Leu348
*3ewl*	−17.65	Zn^+2^, His415, Glu406	Val440, Ile438, Pro437, Ala439, Met345, Leu350
*3kmc*	−11.25	Zn^+2^, Glu406	Met345, Ile438, Pro437, Leu350
*3kme*	−16.42	Zn^+2^, His409, Glu406	Pro437, Ile438, Ala439, Leu348, Met345, Leu350,
*3le9*	−15.23	Zn^+2^, His415, Asp313	Val314, Leu348, Pro437
*3o64*	−13.84	Zn^+2^, His415	Ile394, Leu348, Pro437, Ile438, Val434, Val440, Leu 401, Ala439, Val402
*2i47*	−15.76	Zn^+2^, His415, Glu406	Met345, Leu348, Leu350, Pro437, Ile438, Ala439
*3e8r*	−12.94	Zn^+2^, Glu406	Leu350, Pro437, Ile438, Leu348, Ala439, Met345
*3edz*	−16.72	Zn^+2^, His415, Glu406	Leu350, Met345, Leu348, Ileu438, Ala439, Pro437
*3lgp*	−10.37	Zn^+2^, Glu406	Ala439, Pro437, Ile438, Leu348
*3l0t*	−16.48	Zn^+2^, His409, His415	Leu350, Met345, Leu348, Ala439, Ile438, Pro437
*1bkc*	−17.21	Zn^+2^, His415, Glu406	Met345, Ile438, Leu348, Pro437, Leu350, Ala439
*2oi0*	−11.96	Zn^+2^, His415	Leu350, Leu348, Met345, Ala439, Ile438, Pro437
*3b92*	−16.39	Zn^+2^, His415, His405	Pro437, Ile438, Met345, Val314, Leu350
*3lea*	−14.53	Zn^+2^, His415	Trp312, Met345, Leu384, Val314, Pro437, Ala439, Leu350
*2fv9*	−17.41	Zn^+2^, His415, His405	Leu350, Leu348, Ile438, Ala439, Met345, Pro437
*2fv5*	−16.90	Zn^+2^, His415, His405	Ile438, Leu350, Ala439, Met345, Pro437, Leu348
*3g42*	−15.79	Zn^+2^, His415, His409	Ala439, Met345, Ile438, Leu438, Pro437
*1zxc*	−16.47	Zn^+2^, His405, Glu406	Ile438, Pro437, Leu350, Ala439, Met345, Leu348
*2a8h*	−17.87	Zn^+2^, Glu406, His415, His405	Pro437, Met345, Ile438, Leu348, Ala439, Leu350

## Data Availability

Data are provided within the main text and the Supplementary Materials of article.

## Supplementary Materials

The following are available online at https://www.mdpi.com/article/10.3390/ph14121222/s1, Table S1: The 2D and 3D molecular docking interactions of the docked vitamin D3 with ADAM17 binding site.
