# Supplementary material for: Vitamin D3 Prevents the Deleterious Effects of Testicular Torsion on Testis by Targeting miRNA-145 and ADAM17: In Silico and In Vivo Study
Source: Pharmaceuticals (Basel). 2021 Nov 25;14(12):1222. doi: 10.3390/ph14121222 (PMC8703569; doi:10.3390/ph14121222)
Supplement: Supplementary file 1 [file pharmaceuticals-14-01222-s001.zip › pharmaceuticals-1456981-supplementary.pdf]

Table S1. The 2D and 3D molecular docking interactions of the docked vitamin D3 with ADAM17 binding site.

PDB	2D interactions	3D interactions
2ddf	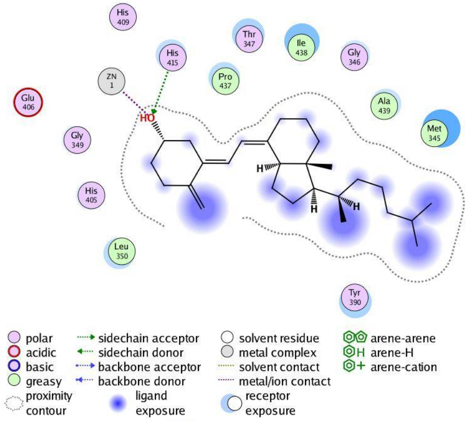	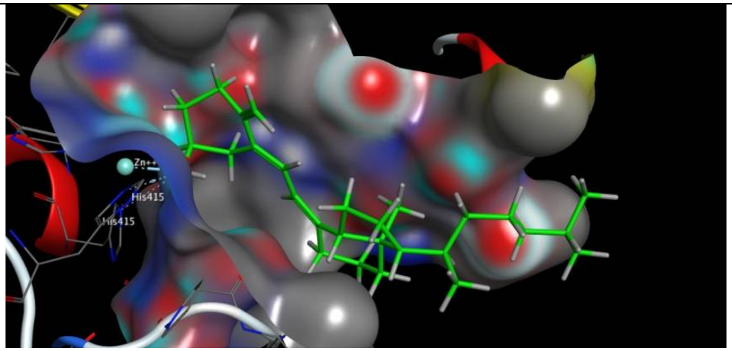
3l0v	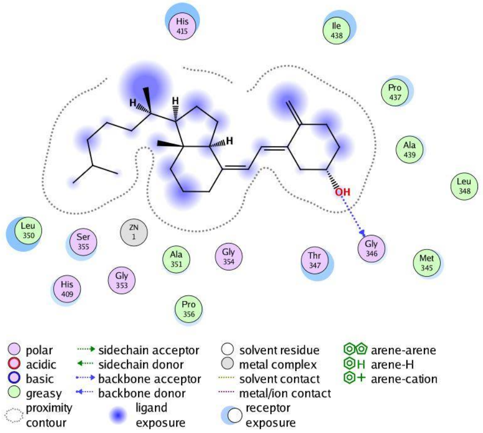	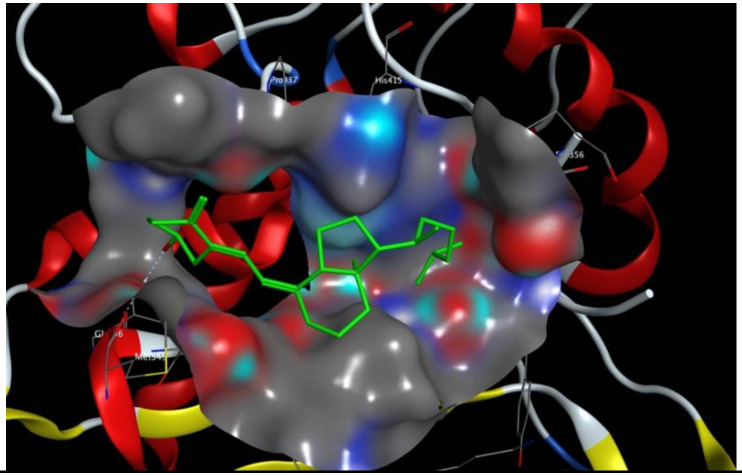

3ewl

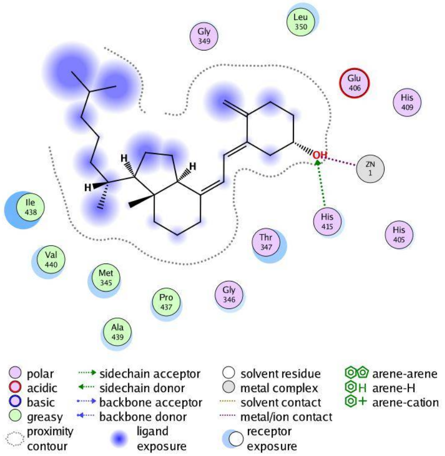
Gly 349
Leu 350
Glu 406
His 409
ZN 1
Ile 438
His 415
His 405
Thr 347
Val 440
Met 345
Gly 346
Pro 437
Ala 439
OH
polar
acidic
basic
greasy
proximity contour
sidechain acceptor
sidechain donor
backbone acceptor
backbone donor
ligand exposure
solvent residue
metal complex
solvent contact
metal/ion contact
receptor exposure
arene-arene
arene-H
arene-cation

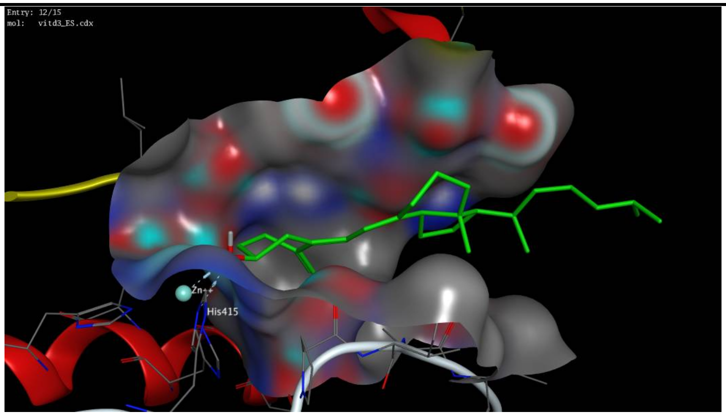
Entry: 12/15
mol: vitd3_ES.cdx
Zn++
His415

3kmc

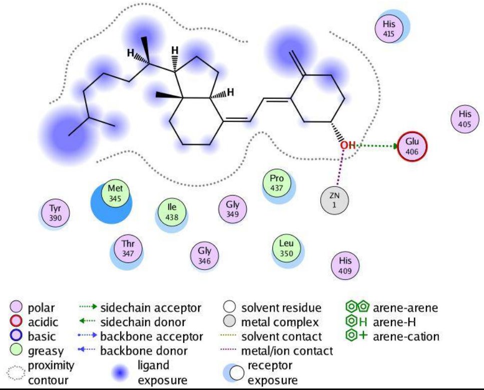
His 415
His 405
Glu 406
ZN 1
OH
Tyr 390
Met 345
Ile 438
Gly 349
Pro 437
Thr 347
Gly 346
Leu 350
His 409
polar
acidic
basic
greasy
proximity contour
sidechain acceptor
sidechain donor
backbone acceptor
backbone donor
ligand exposure
solvent residue
metal complex
solvent contact
metal/ion contact
receptor exposure
arene-arene
arene-H
arene-cation

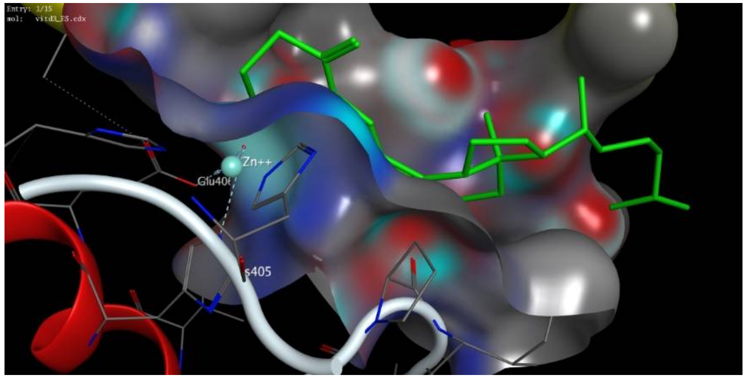
Zn++

3kme

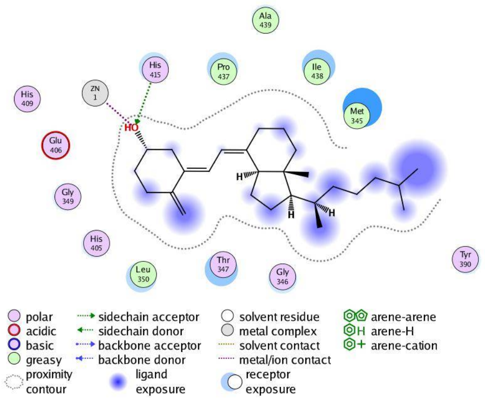

Ala 439
His 415
Pro 437
Ile 438
ZN 1
His 409
Met 345
HO
Glu 406
Gly 349
His 405
Leu 350
Thr 347
Gly 346
Tyr 390
polar
acidic
basic
greasy
proximity contour
sidechain acceptor
sidechain donor
backbone acceptor
backbone donor
ligand exposure
solvent residue
metal complex
solvent contact
metal/ion contact
receptor exposure
arene-arene
arene-H
arene-cation

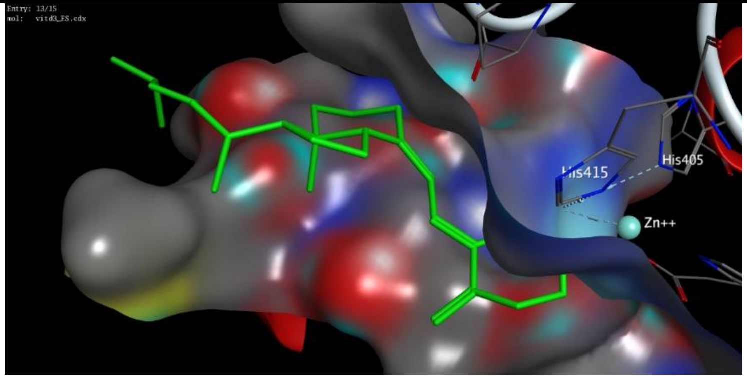

His405
Zn++

3le9

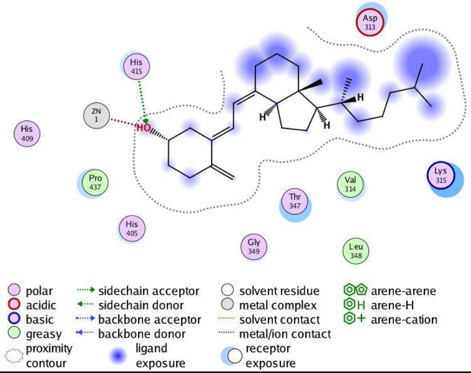

Asp 313
His 415
ZN 1
HO
His 409
Pro 437
Lys 315
Thr 347
Val 314
His 405
Gly 349
Leu 348
polar
acidic
basic
greasy
proximity contour
sidechain acceptor
sidechain donor
backbone acceptor
backbone donor
ligand exposure
solvent residue
metal complex
solvent contact
metal/ion contact
receptor exposure
arene-arene
arene-H
arene-cation

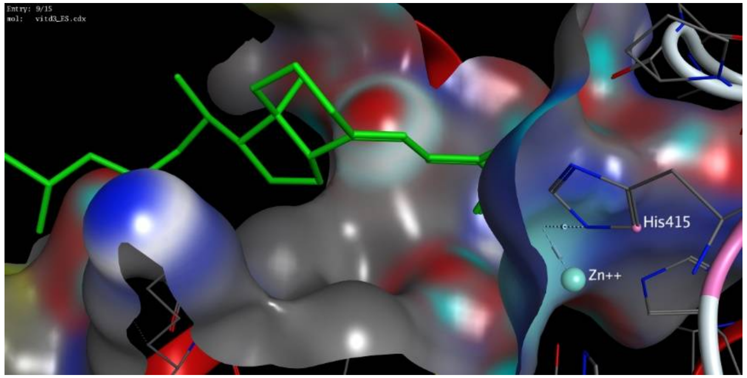

His415
Zn++

3o64

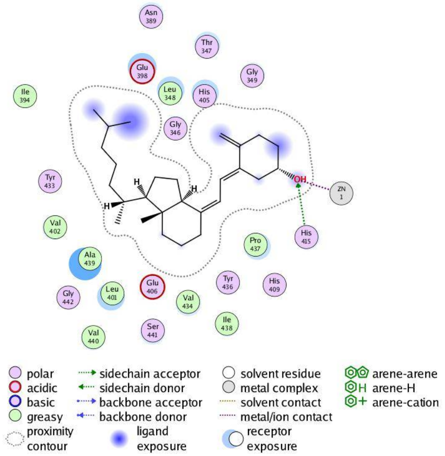
polar
acidic
basic
greasy
proximity contour
sidechain acceptor
sidechain donor
backbone acceptor
backbone donor
ligand exposure
solvent residue
metal complex
solvent contact
metal/ion contact
receptor exposure
arene-arene
arene-H
arene-cation

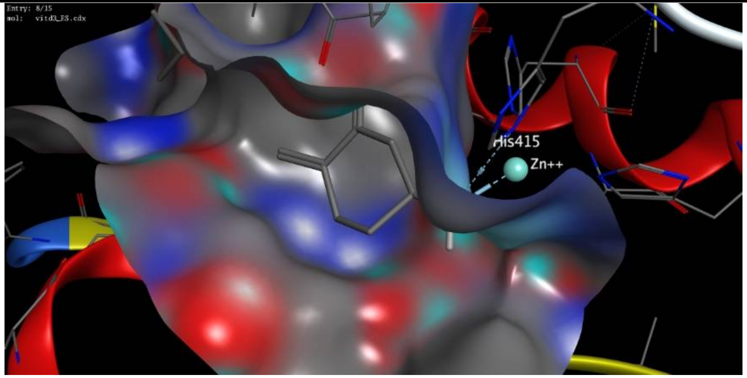
His415
Zn++

2i47

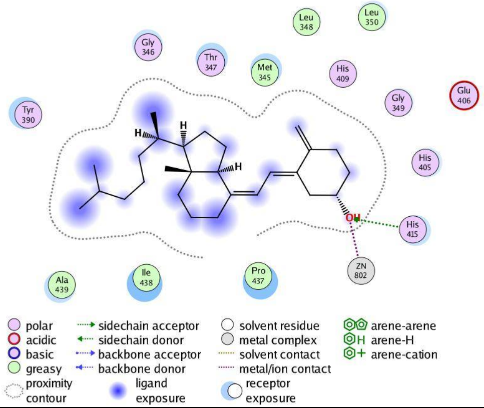
polar
acidic
basic
greasy
proximity contour
sidechain acceptor
sidechain donor
backbone acceptor
backbone donor
ligand exposure
solvent residue
metal complex
solvent contact
metal/ion contact
receptor exposure
arene-arene
arene-H
arene-cation

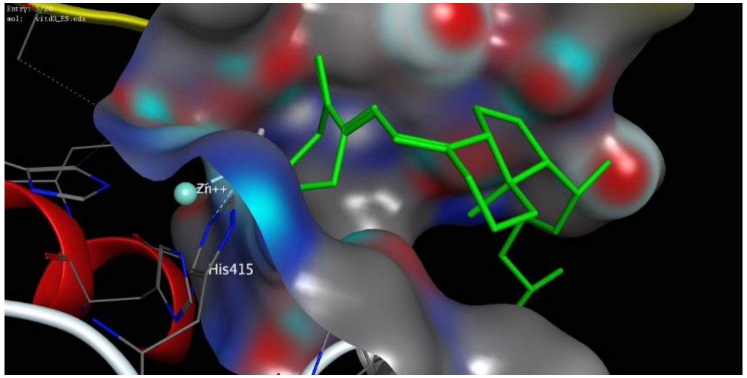
Zn++
His415

3e8r

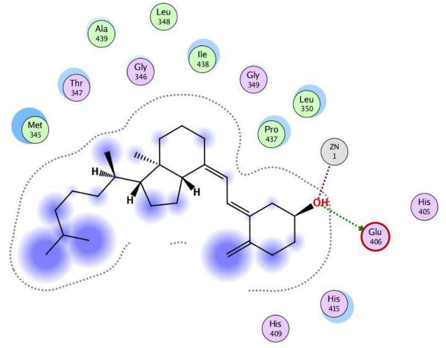

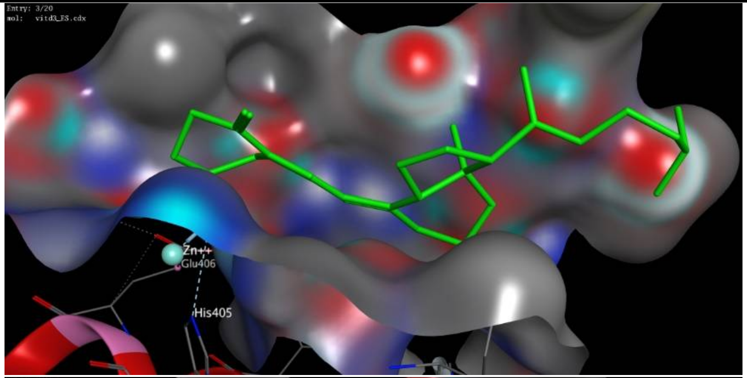

3edz

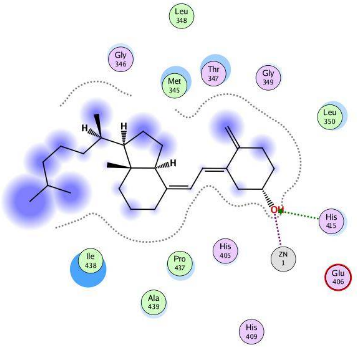

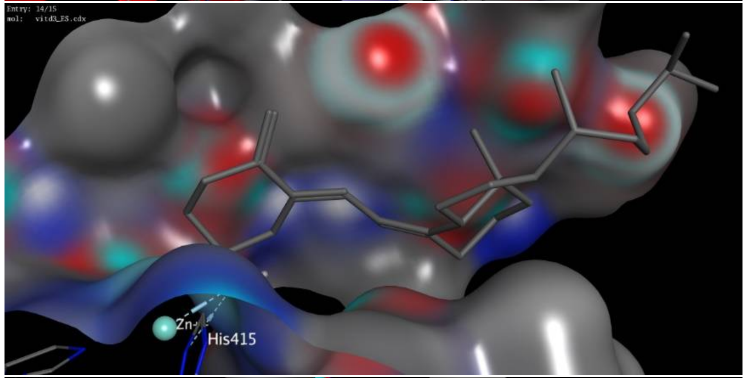

3lgp

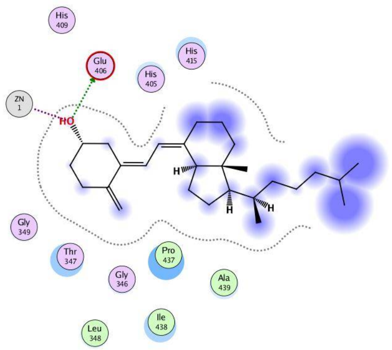

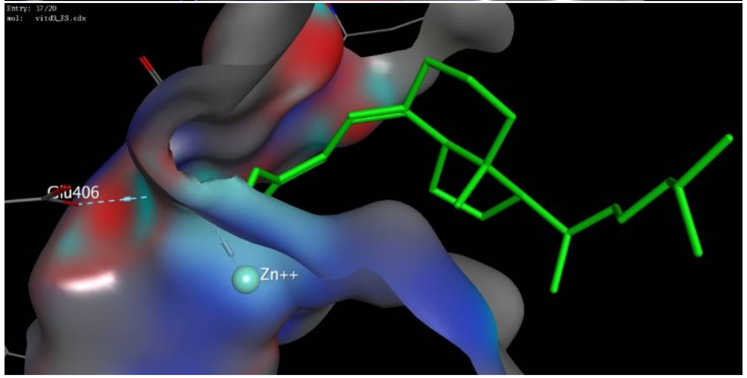

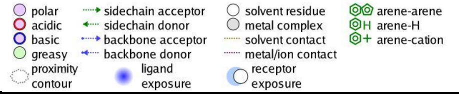

1bkc

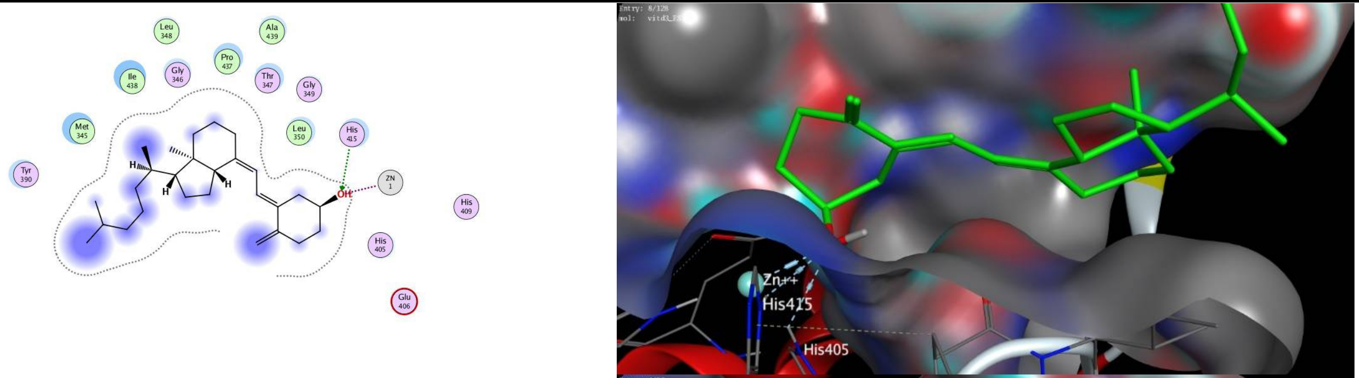

2oi0

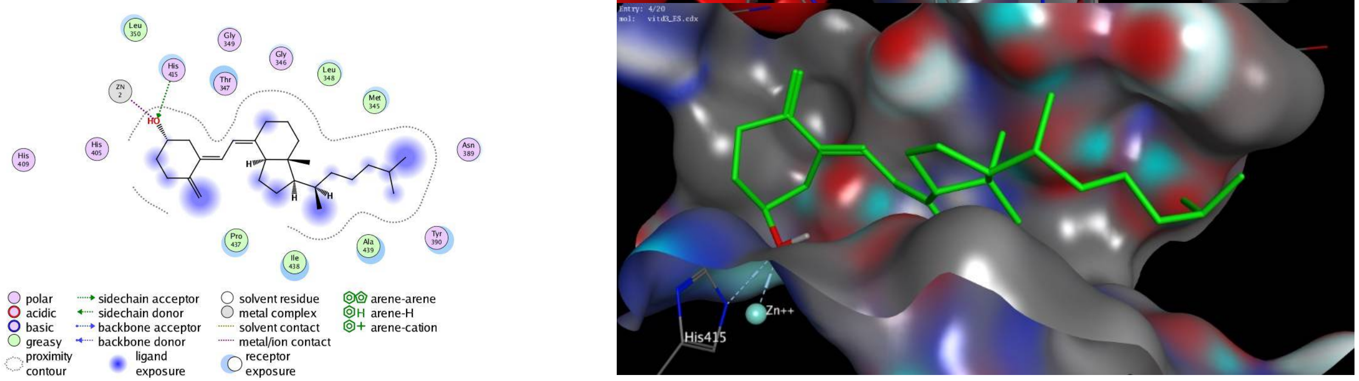

3lea

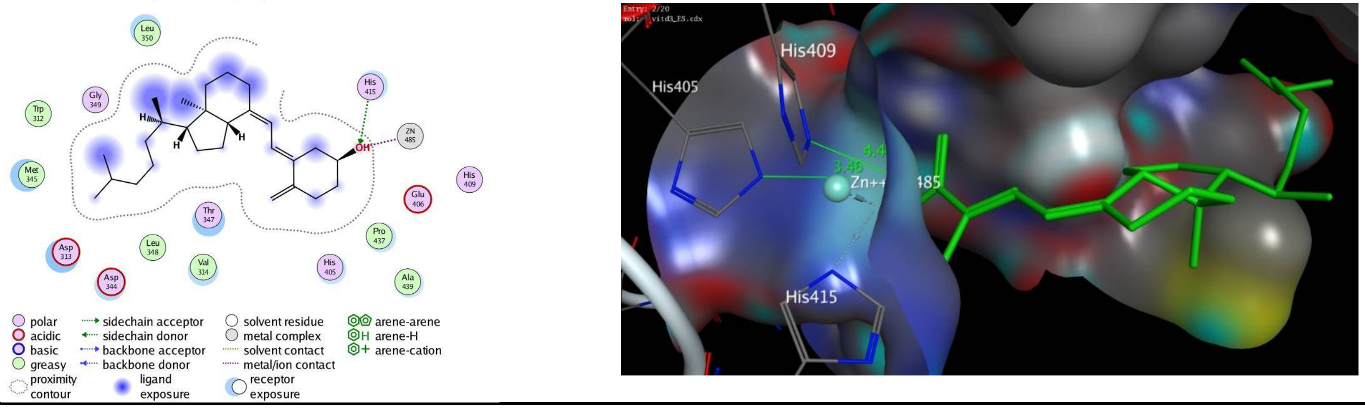

2fv9

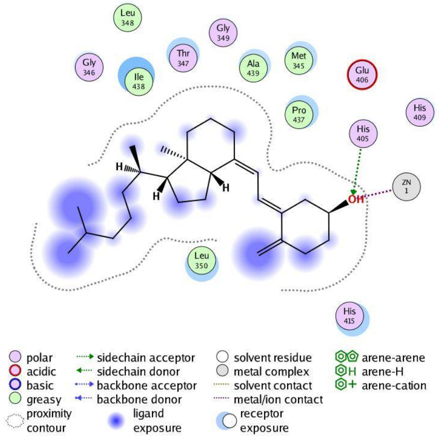
Leu 348
Gly 349
Gly 346
Thr 347
Ile 438
Ala 439
Met 345
Glu 406
Pro 437
His 409
His 405
ZN 1
OH
Leu 350
His 415
polar
acidic
basic
greasy
proximity contour
sidechain acceptor
sidechain donor
backbone acceptor
backbone donor
ligand exposure
solvent residue
metal complex
solvent contact
metal/ion contact
receptor exposure
arene-arene
arene-H
arene-cation

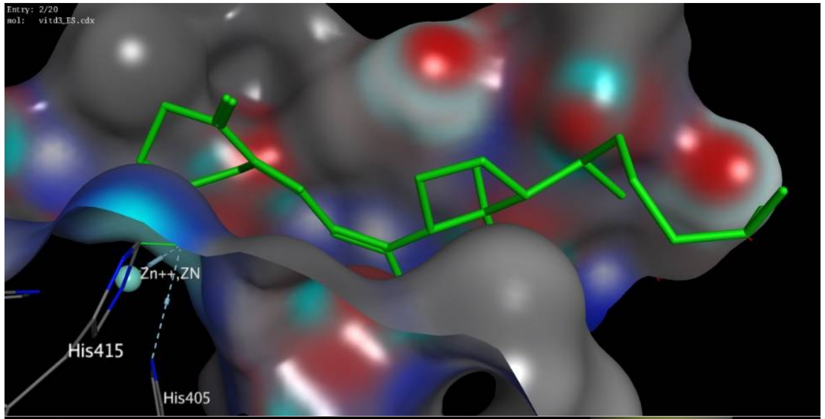
Zn++,ZN
His415
His405

2fv5

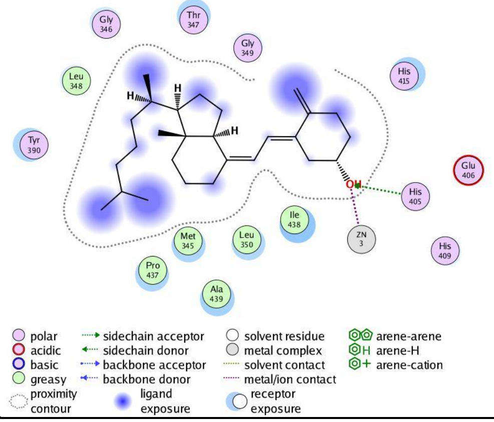
Gly 346
Thr 347
Gly 349
Leu 348
His 415
Tyr 390
OH
Glu 406
His 405
Ile 438
Met 345
Leu 350
ZN 3
His 409
Pro 437
Ala 439
polar
acidic
basic
greasy
proximity contour
sidechain acceptor
sidechain donor
backbone acceptor
backbone donor
ligand exposure
solvent residue
metal complex
solvent contact
metal/ion contact
receptor exposure
arene-arene
arene-H
arene-cation

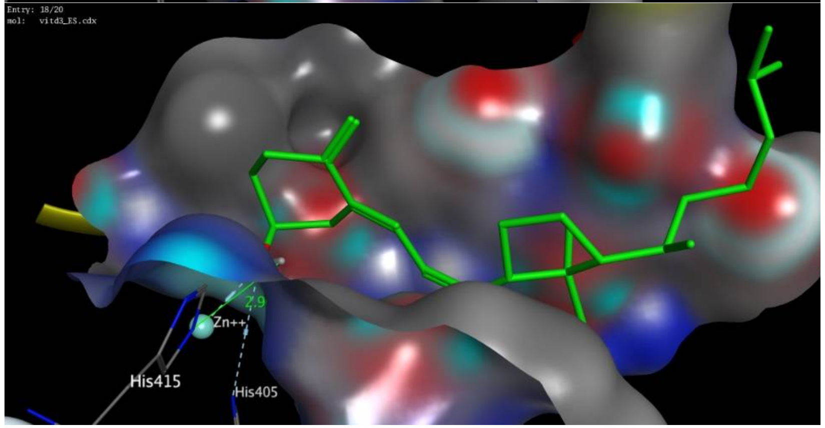
2.9
Zn++
His415
His405

3g42

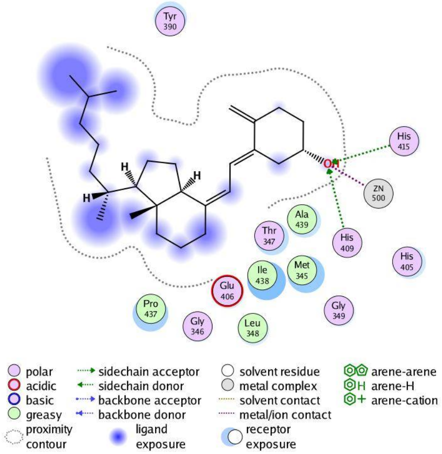
Tyr 390
His 415
ZN 500
Ala 439
Thr 347
His 409
His 405
Ile 438
Met 345
Glu 406
Pro 437
Gly 346
Leu 348
Gly 349
polar
acidic
basic
greasy
proximity contour
sidechain acceptor
sidechain donor
backbone acceptor
backbone donor
ligand exposure
solvent residue
metal complex
solvent contact
metal/ion contact
receptor exposure
arene-arene
arene-H
arene-cation

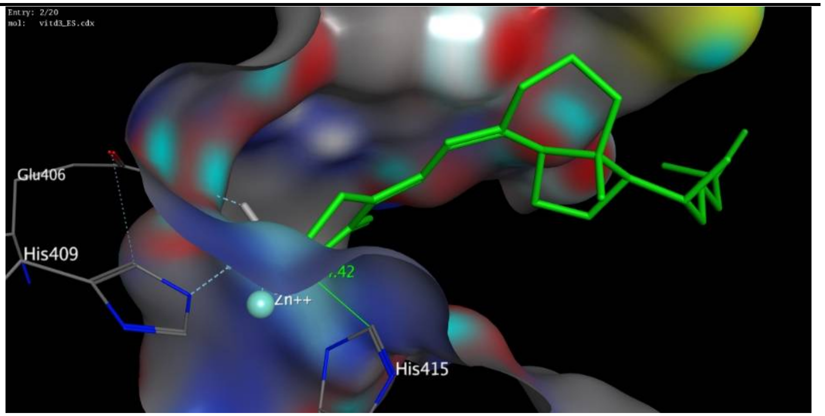
Glu406
His409
Zn++
His415

2a8h

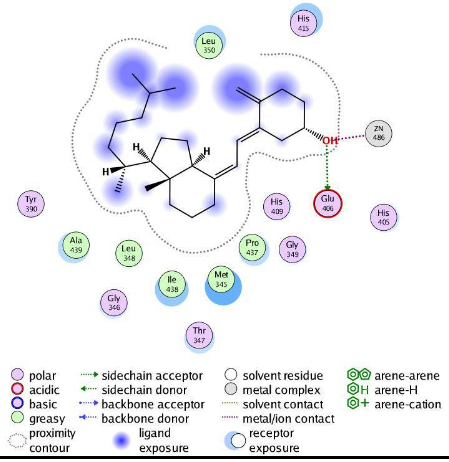
His 415
Leu 350
ZN 486
Tyr 390
Glu 406
His 409
His 405
Ala 439
Leu 348
Pro 437
Gly 349
Ile 438
Met 345
Gly 346
Thr 347
polar
acidic
basic
greasy
proximity contour
sidechain acceptor
sidechain donor
backbone acceptor
backbone donor
ligand exposure
solvent residue
metal complex
solvent contact
metal/ion contact
receptor exposure
arene-arene
arene-H
arene-cation

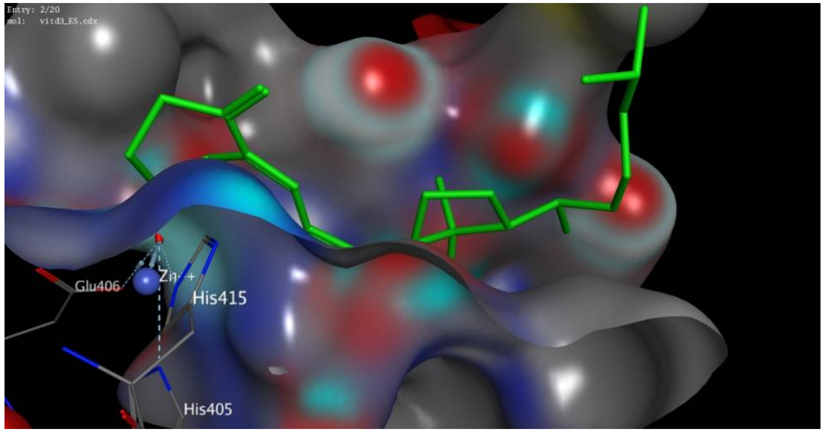
Glu406
His415
His405
